# An Atomistic View on the Mechanism of Diatom Peptide‐Guided Biomimetic Silica Formation

**DOI:** 10.1002/advs.202401239

**Published:** 2024-06-14

**Authors:** Fanny Kozak, Dörte Brandis, Christopher Pötzl, Ludovica M. Epasto, Daniela Reichinger, Dominik Obrist, Herwig Peterlik, Anton Polyansky, Bojan Zagrovic, Fabian Daus, Armin Geyer, Christian FW Becker, Dennis Kurzbach

**Affiliations:** ^1^ Institute of Biological Chemistry, Faculty of Chemistry University of Vienna Währinger Str. 38 Vienna 109 Austria; ^2^ Vienna Doctoral School in Chemistry (DoSChem) University of Vienna Währinger Str. 42 Vienna 1090 Austria; ^3^ Faculty of Physics University of Vienna Boltzmanngasse 5 Vienna 1090 Austria; ^4^ Department of Structural and Computational Biology Max Perutz Labs University of Vienna Campus Vienna Biocenter 5 Vienna A‐1030 Austria; ^5^ Faculty of Chemistry Philipps‐Universität Marburg 35032 Marburg Germany

**Keywords:** diatom peptides, fractal cluster formalism, molecular dynamics simulations, NMR spectroscopy, peptide‐guided mineralization, silica templating

## Abstract

Deciphering nature's remarkable way of encoding functions in its biominerals holds the potential to enable the rational development of nature‐inspired materials with tailored properties. However, the complex processes that convert solution‐state precursors into solid biomaterials remain largely unknown. In this study, an unconventional approach is presented to characterize these precursors for the diatom‐derived peptides R5 and synthetic Silaffin‐1A_1_ *(syn*Sil‐1A_1_). These molecules can form defined supramolecular assemblies in solution, which act as templates for solid silica structures. Using a tailored structural biology toolbox, the structure‐function relationships of these self‐assemblies are unveiled. NMR‐derived constraints are employed to enable a recently developed fractal‐cluster formalism and then reveal the architecture of the peptide assemblies in atomistic detail. Finally, by monitoring the self‐assembly activities during silica formation at simultaneous high temporal and residue resolution using real‐time spectroscopy, the mechanism is elucidated underlying template‐driven silica formation. Thus, it is demonstrated how to exercise morphology control over bioinorganic solids by manipulating the template architectures. It is found that the morphology of the templates is translated into the shape of bioinorganic particles via a mechanism that includes silica nucleation on the solution‐state complexes’ surfaces followed by complete surface coating and particle precipitation.

## Graphical abstract

Using the diatom‐derived peptides R5 and synthetic Silaffin‐1A_1_, the mechanism is elucidated underlying template‐driven silica formation and it is demonstrated how to exercise morphology control over bioinorganic solids by manipulating the template architectures. The morphologies of self‐assembled peptide templates are translated into the shape of bioinorganic particles by silica nucleation on the solution‐state complexes’ surfaces followed by complete surface coating.

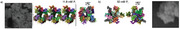

## 1 Introduction

Today, still, the most advanced functional materials are those found in nature. Among them, biominerals, i.e., solid phases formed by living organisms, constitute an extensive array of composites with superior functionality ranging from the stability of bones^[^ ^1^, ^2^, ^3^ ^]^ to the efficiency of iron storage^[^ ^3^, ^4^ ^]^ to magnetic field and gravity sensing.^[^ ^5^ ^]^ Biomimetic materials’ design inspired by biominerals, thus, offers excellent potential for creating ecologically benign materials with superior properties. It promises solutions in many pressing research areas, from energy storage to vaccine delivery.^[^ ^6^, ^7^, ^8^ ^]^ Significant advances, such as superhydrophobic surfaces or enzyme and antigen encapsulation,^[^ ^9^, ^10^ ^]^ already showcase this potential. However, genuine control over biomineralization pathways, which would enable rational biomimetic materials design,^[^ ^11^ ^]^ has not been achieved yet, not least due to the complexity of these processes and the resulting challenges for their experimental characterization.

In this regard, silica‐precipitating peptides derived from diatoms are particularly intriguing as these can act as templates for highly defined nanostructures under mild conditions, two desirable aspects with respect to biomimetic materials’ design.^[^ ^12^, ^13^ ^]^ In‐vivo peptide‐driven silica formation is a complex process promoted by specific SDV (silica deposition vesicles), which host the mineralization event.^[^ ^13^, ^14^ ^]^ This behavior of microemulsion and micellar organization has been proposed to play a role in diatom cell wall biogenesis, too.^[^ ^15^ ^]^

In‐vitro biomimetic silica formation is typically guided by supramolecular assembly of the peptides into template structures.^[^ ^14^, ^16^, ^17^ ^]^ Such events are often triggered by multivalent counterions, which act as ion bridges between the peptide units (for some conditions, such complexes are also referred to as polyelectrolyte coacervates, PEC).^[^ ^18^, ^19^, ^20^ ^]^ Interestingly, for purely inorganic systems, such as calcium phosphates and carbonates, comparable templating mechanisms have recently been reported based on so‐called pre‐nucleation clusters (PNC) forming in the absence of any complex biomolecule.^[^ ^16^, ^21^, ^22^, ^23^, ^24^, ^25^, ^26^, ^27^, ^28^, ^29^, ^30^, ^31^, ^32^, ^33^, ^34^, ^35^, ^36^, ^37^, ^38^, ^39^, ^40^ ^]^ Only for such “minimal systems” were the precursor structures determined so far.

Strikingly though, naturally occurring combined peptide‐ and inorganic ion‐based solution‐state precursors as in peptide‐guided biomineralization^[^ ^40^, ^41^, ^42^, ^43^ ^]^ have almost entirely evaded structure‐function determination so far – not least due to a lack of methodology to access these highly complex species.^[^ ^21^ ^]^

Yet, to truly rationalize and exploit the full potential of biomimetic materials, a detailed functional characterization of such template complexes is essential since their structural dynamics predetermine the morphologies and functions of the solid minerals resulting from biomineralization processes. The underlying structure‐activity relationships (SAR) must be understood when aiming to control processes for creating tailored materials, hence warranting deeper investigations.

In general, in peptide‐guided mineralization based on self‐assembling template structures,^[^ ^44^ ^]^ many mechanistic details could already be deciphered. However, further research is needed to deeply understand the mineralization mechanism and expand applications, with potential for advancements, e.g., through machine learning‐guided peptide design and molecular‐level investigations.^[^ ^44^ ^]^

Herein, we report a methodology to help tackle this challenge. It contains several innovative aspects, such as real‐time nuclear magnetic resonance (NMR) spectroscopy to monitor silica formation events and 2D relaxation NMR of phosphorylation sites for high‐resolution NMR of post‐translationally modified peptides. Integration of NMR‐derived structural information of the peptides in solution with novel “fractal cluster” molecular dynamics (MD) simulations^[^ ^45^ ^]^ and electron microscopy (EM) then allowed us to characterize the structure and dynamics of peptide at atomistic detail even for high molecular weights and track the material formation events from the solution‐state template to the solid state at simultaneously high spatial and temporal resolution.

**Figure 1:**
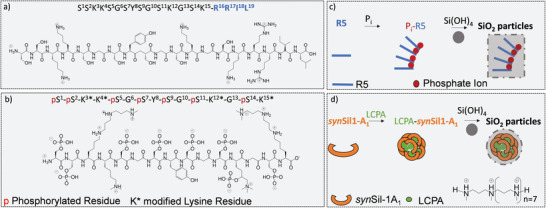
Interaction steps, primary sequences, and structures of the biomimetic silica‐precipitating systems R5 and *syn*Sil‐1A_1_. a) The sequence of peptide R5. The peptide is stripped of all PTMs. Only the primary sequence mimics the natural *nat*Sil‐1A_1_ peptide. The structure corresponds to the average side‐chain protonation state at the experimental pH of 6.5. b) The synthetic variant *syn*Sil‐1A_1,_ which lacks the RRIL motif of R5 but bears all naturally occurring PTMs, albeit somewhat shorter lysine LCPA modifications. c,d) Simplified visualization of major steps of the herein‐studied silicification processes.

We employ these capacities to investigate two silica‐precipitating peptides derived from diatoms: a synthetic variant of fully post‐translationally modified, naturally occurring *nat*Sil‐1A_1_ (*syn*Sil‐1A_1_)^[^ ^20^ ^]^ and the peptide R5,^[^ ^46^, ^47^ ^]^ a version of *nat*Sil‐1A_1_ not containing any posttranslational modifications (PTMs), but an additional C‐terminal RRIL motif (**Figure** **1**a,b shows the peptides’ structures and Figure 1c,d visualizes, in a simplified manner, the major steps of the peptide assembly process and its involvement in the silification process). R5 and *syn*Sil‐1A_1_ both self‐assemble into templates for silica deposition upon neutralization of their side‐chain charges.

Peptide sidechain protonation is, thus, essential for the function of the two studied peptides. At our experimental pH of 6.5, considering the high buffer strength and assuming the absence of any complex local environments (which may alter pK_a_ values), all lysines, amines, and arginines are fully protonated.^[^ ^48^ ^]^ At the same time, the phosphoserines are (on average) singly protonated.^[^ ^49^ ^]^ For the present study, we used inorganic phosphate (P_i_; the abbreviation is herein understood to represent all species involved in the HPO_4_^2−^ / H_2_PO_4_^−^ equilibrium) or long‐chain polyamines (LCPA), respectively, as counterions.

The resulting supramolecular structures initiate silica formation on their surfaces to yield highly defined solid phases under mild aqueous conditions upon exposure to only millimolar silicate concentrations^[^ ^20^, ^46^, ^50^, ^51^ ^]^ – valuable features for biomimetic materials design.

We report two key findings for both peptides:

- The template assemblies trigger a two‐step silica co‐precipitation mechanism: silica nucleation on their surfaces, followed by completion and rigidification of the surface coating. As a result, the template size predetermines the volume of the resulting silica precipitates, *ceteris paribus*, i.e., for given peptide and ion concentrations, as well as speciation states.
- The peptide self‐assemblies are fractal objects with repeats of tripeptide clusters as basic building blocks, whose dimensionality and surface structures orchestrate the shape of the biomimetic silica nanoparticles.

In other words, the peptide self‐assemblies can be understood as large colloids constituted by small trimeric building blocks with similar structures independent of the observed length scales, i.e., as fractals.^[^ ^52^ ^]^ These objects can, therefore, be comprehensively characterized by their overall size as well as fractal dimension *d_f_* (e.g., rods, discs, or spheres),^[^ ^53^, ^54^, ^55^ ^]^ which in return determine the surface available for silica coating.

These findings allow for the prediction and rationalization of the solid‐state morphology of bioinorganic silica nanoparticles and the exploitation of the self‐assemblies’ templating functions.

## 2 Results and Discussion

The peptide R5 comprises 19 amino acids, including a C‐terminal RRIL motif (Figure 1a).^[^ ^47^ ^]^ The employed *syn*Sil‐1A_1_ comprises 15 amino acids (without the RRIL motif), resembling the natively occurring fully matured version of the R5 peptide. In this peptide, all serine residues are phosphorylated, and all lysine residues are heavily modified either with oligo‐propylene imine, phosphocholine, or by methylation^[^ ^20^ ^]^ (Figure 1b).

R5 is a peptide often used in biotechnological applications. In contrast, *nat*Sil‐1A_1_ is a naturally occurring peptide. Both peptides share a similar primary sequence (apart from the RRIL motif) and are, hence, ideally suited for a comparison of the silification processes they induce. Concerning the counterions, due to the lack of phosphorylation sites in R5, the addition of negative charges via P_i_ is often necessary for efficient self‐assembly. With respect to *nat*Sil‐1A_1_, its synthetic analog *syn*Sil‐1A_1_ features shorter LCPA side‐chain modification, such that additional unbound LCPA needs to be present for efficient self‐assembly.

### 2.1 Assembly Analysis by Residue‐Resolved NMR

To access the supramolecular structural dynamics of R5 and *syn*Sil‐1A_1_ assemblies in the presence of phosphate or LCPA, respectively (for details on the employed LCPA, see reference^[^ ^20^ ^]^), we devised heteronuclear NMR correlation experiments detecting either ^1^H‐^15^N or ^13^C‐^15^N backbone signals or unconventional ^1^H‐^31^P phosphorylation site resonances.

#### 2.1.1 R5 Peptide

**Figure 2:**
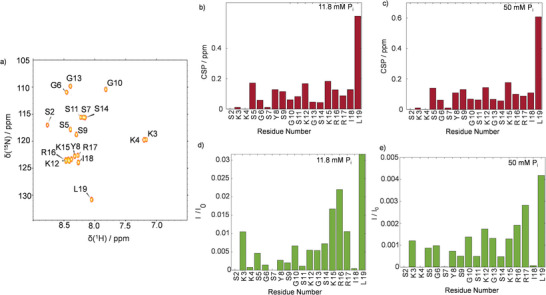
a) ^1^H‐^15^N HSQC of R5 with signal assignments as indicated. b,c) CSP upon P_i_ exposure at concentrations of 11.8 and 50 mm, respectively. d,e) Intensity ratios (*I*/*I*_0_) between resonances in the presence (*I*) and absence (*I*_0_) of 11.8 or 50 mm P_i_. Residues 2, 4, and 7 remained below the detection threshold in the presence of phosphate. Hence, the respective values are missing.

The R5 peptide exists as an intrinsically disordered monomer in aqueous solutions with a propensity to transiently form a central β‐turn under the herein probed experimental conditions (vide infra).^[^ ^38^, ^47^, ^56^ ^]^ Upon dissolution in a phosphate‐buffered saline (PBS) at pH 6.5, the monomers assemble into large supramolecular complexes that act as templates for silica precipitation.^[^ ^57^, ^58^ ^]^ To decipher their structural dynamics, we expressed isotopically enriched R5, as detailed in the experimental section. Then, we recorded ^1^H‐^15^N HSQC (heteronuclear single quantum coherence) spectra (**Figure** **2**a; Figure S11, Supporting Information) of the peptide backbone amides to obtain residue‐resolved chemical shift perturbations (CSP) and intensity changes upon self‐assembly at varying P_i_ concentrations (11.8 or 50 mm). While the CSP reports on local changes in the chemical environment, the intensities report signal losses through reduced mobility or chemical exchange.

Figure 2b,c show that CSP was observed throughout the entire primary sequence upon P_i_‐exposure with particularly strong effects at the C‐terminal residue L19. Interestingly, the observed CSP patterns were similar for low and high P_i_ concentrations (11.8 and 50 mm). This observation points towards a constant R5 conformation independent of the solution conditions.

Upon self‐assembly, we further observed that signal amplitudes reduced non‐uniformly. In particular, the C‐terminal residues 15–19 showed reduced intensity losses compared to the rest of the R5 peptide (Figure 2d,e).

While the overall profiles are to large degrees similar, small yet significant differences can nevertheless be observed, particularly for residues 15–18 (Figure 2; Figure S1, Supporting Information). This finding is well in line with our simulation results, which show that the phosphate counterions bind differently to the RRIL motif under the probed conditions (Figure S2, Supporting Information; at 11.8 mm phosphate, a weaker coordinated species is observed than at 50 mm). Hence, differing intensity profiles can be expected.

The NMR data imply that more peptides take part in the template formation at high (counter‐)ion strength, even though self‐assembly was clearly observed under all probed conditions. However, the local structural adaptions upon self‐assembly are P_i_ concentration‐independent as suggested by the CSP. ^15^N transverse relaxation rates *R*_2_ confirm this interpretation by significant increase upon P_i_ exposure, which points toward more restricted peptide backbone mobility upon self‐assembly (Figures S3–S6, Supporting Information). Similar behavior has recently been described for other RRIL‐carrying peptide self‐assemblies^[^ ^36^ ^]^: The NMR‐detectible solute species are in exchange with large self‐assemblies upon P_i_‐exposure, and the C‐terminal RRIL residues form the solvent‐accessible surfaces (SAS) of the self‐assemblies. As a result, they retain more flexibility than core residues and, thus, have stronger signal amplitudes. In other words, the surface residues lead to sharper (exchange‐averaged) signals, while the core residues are broadened beyond the detection threshold.

**Figure 3:**
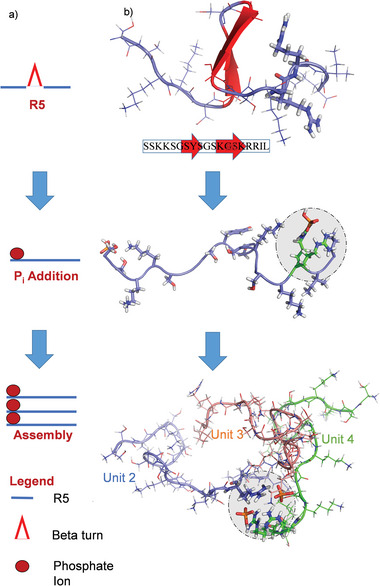
Phosphate interaction and supramolecular architecture of R5 assemblies. a) Scheme depicting the tripeptide assembly mechanism as observed in the MD simulations. b) Snapshots, each after 1 µs of an MD simulation, of three MD runs. From top to bottom: R5 in water, R5 in the presence of P_i_, and self‐assembly of three R5 units in the presence of P_i_. The snapshots visualize the tripeptide assembly process by binding of P_i_ to the RRIL motifs of R5. Statistical analysis of the simulations and ^31^P NMR spectra of the P_i_ ions can be found in Figure S7 (Supporting Information).

Judging from the average residual NMR signal intensities, ≈0.7 and ≈0.01% of the peptides remained outside the large self‐assemblies at P_i_ concentrations of 11.8 and 50 mm, respectively. Importantly, when employing ^13^C direct‐detected NMR experiments, which also resolve the core residues within the self‐assemblies, as recently shown by Forman‐Kay and co‐workers,^[^ ^59^ ^]^ we were able to prove that the structure of R5 within the self‐assemblies is conserved and independent of the P_i_ concentration even local mobility decreases (Figures S10–S12, Supporting Information). To rationalize the NMR data and elucidate the structure of the R5 peptides in solution and the assemblies, we analyzed our system by all‐atom, explicit‐solvent MD simulations (for details, see the Experimental Section and the Supporting Information). We found that neat R5 remained mostly unfolded in solution. It transiently adopted a β‐turn‐like structure (**Figure** **3** top) under the probed conditions. The energy‐minimized structure of the monomer was then further equilibrated in the presence of P_i_. This indicated that phosphate binds to the two arginine residues within the RRIL motif of R5 units within ca. 200 ns and leads to an expansion of the peptide backbone (Figure 3 center; Figures S2 and S7–S9, Supporting Information). Direct phosphate binding was further confirmed by ^31^P NMR (Figure S6, Supporting Information). Once the simulations had reached a “plateau” in terms of RMSD from the starting structure, these complexes remained stable (see the Figure S6, Supporting Information). However, it should be noted that the third R5 unit was less strongly correlated according to a dynamic cross‐correlation analysis. Hence, while the main species observed after equilibration were trimers in the simulation, an exchange between dimeric and trimeric forms cannot be excluded.

In the next step, MD simulations of four copies of the resulting R5/P_i_ complex in solution were carried out in the presence of 11.8 or 50 mm P_i_. For both conditions, we found that Arg‐P_i_‐Arg bridges are responsible for a supramolecular assembly of R5 (Figure 3 bottom) into tripeptide units. The RRIL motif functioned as a bidirectional phosphate trap and linker between two peptides – a mechanism that potentially compensates for the lack of phosphorylation in non‐post‐translationally modified R5 to trigger self‐assembly.^[^ ^46^, ^50^, ^60^, ^61^ ^]^ In particular, residues R16 and R17 led to the observation of a direct and constant phosphate binding mode (see Figure S7, Supporting Information). The exposed N‐terminal amino acids of R5 constituted a further interaction site via the N‐terminal SKKS motif between residues 2 and 5 (Figure 3b). This motif formed a second contact point. Residue K3 and K4 showed a strong direct phosphate interaction (see Figure S2, Supporting Information) similar to that of the arginines within the RRIL motif.

The tripeptide structures found in the MD simulations were then experimentally confirmed by diffusion‐ordered NMR spectroscopy (DOSY) experiments. We found an experimental hydrodynamic radius *R_h_* of 0.8±0.1 nm and 1.3±0.1 nm for R5 in the absence and presence of P_i_, respectively. The simulations showed an *R_h_* of 0.8±0.3 nm and 1.3±0.4 nm, respectively (Figure S8, Supporting Information), agreeing closely with the experimental data. Furthermore, the sizes of the peptides were confirmed independently by small‐angle X‐ray scattering (SAXS) experiments (see Figures S8 and S9, Supporting Information). The approximately three‐fold volume increase upon assembly confirmed an aggregate volume that fits three R5 units, and these findings further agree with earlier studies showing that R5 assemblies are constituted by repeats of subunits with 3 densely packed R5 units that further assemble into larger oligomers in a second step in solution.^[^ ^58^, ^61^, ^62^, ^63^ ^]^

Note that the employed *R_h_*‐values of the trimers are different from those of dimers (see the Figures S13 and S14, Supporting Information), hence corroborating the notion that a majority of trimers are involved in the formation of larger assemblies. However, small populations of dimers (as, e.g., studied by Pfaendner and co‐workers^[^ ^37^ ^]^) in equilibrium with trimers cannot be excluded.

Note that for similar phosphate concentrations as probed here, hydrodynamic radii of 200–250 nm have been determined for R5 assemblies by static light scattering (SLS) in combination with SAXS experiments.^[^ ^58^ ^]^ Such a size corresponds to molecular weights of >>100 kDa. In this size range ^1^H‐^15^N cross‐peaks are heavily broadened, typically beyond the detection threshold, which is in line with the intensity losses in Figure 2. Consequently, the residues that remained detectible by ^1^H DOSY (see Figures S8 and S9, Supporting Information), report and *R_h_* referring only to those tripeptide units, not bound in larger “supra‐assemblies”.

Relating the NMR and MD data, we so far conclude that the peptide self‐assemblies comprise structure‐conserving repeats of subunits made of three R5 molecules connected by phosphate‐based salt bridges. All large structures formed under these conditions exceed the sensitivity of the recorded HSQC and DOSY experiments, and only free tripeptide units contributed to the remaining signals. Increasing the availability of P_i_ ions thereby leads to a larger number of tripeptide units bound in the supramolecular structures and to loss of signal intensity.

#### 2.1.2 synSil‐1A_1_

To further probe our approach and consolidate our findings, we compared R5 to *syn*Sil‐1A_1_ (Figure 1b), assessing whether the mechanism observed is also reflected in substrates closely resembling the native, fully modified *nat*Sil‐1A_1_ of *C. fusiformis*. Due to the challenging synthesis^[^ ^20^ ^]^ of *syn*Sil‐1A_1_, it was not ^13^C/^15^N‐enriched. Hence, to achieve residue resolution, we adapted the ^1^H‐^31^P HSQC, devised initially by Marino and Luy^[^ ^64^ ^]^ for DNAs, to the phosphorylated side chains. We combined this pulse sequence with a Carr‐Purcel‐Meiboom‐Gil (CPMG) block to assess CSP as well as ^31^P‐*R*_2_ rates upon self‐assembly (see Figures S15–S20, Supporting Information). ^1^H‐^31^P HSQC of the phosphorylation sites enables multidimensional NMR access, providing an alternative for high‐resolution studies of hyperphosphorylated peptides and proteins without the need for ^13^C and/or ^15^N isotope enrichment.

**Figure 4:**
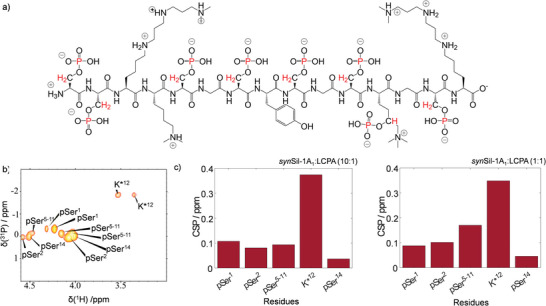
NMR fingerprints of *syn*Sil‐1A_1_ self‐assembly. a) *syn*Sil‐1A_1_ structure and nuclei correlated in the NMR experiments (red). b)^1^H‐^31^P HSQC of *syn*Sil‐1A_1_ phosphorylation sites. The signal assignment is indicated c) CSP upon LCPA exposure at 0.1 and 1 molar equivalents of LCPA.

**Figure** **4**a visualizes the correlated nuclei in *syn*Sil‐1A_1_, and Figure 4b shows a representative ^1^H‐^31^P HSQC phosphorylation site spectrum. The corresponding CSP at varying LCPA counterion concentrations (*syn*Sil‐1A_1_:LCPA = 10:1 and 1:1) are shown in Figure 4c.

Note that the utilized LCPA featured a total of 10 nitrogen atoms linked by propyl linkers. For the methylated LCPA variant employed in our research,^[^ ^20^ ^]^ the pKa values are 10.6 for the complete protonation of terminal secondary amines and 9.6 for the central tertiary amines.^[^ ^65^ ^]^ Consequently, we presume all amines to be fully protonated (aligning with the protonation degree predicted by our modeling routines; see the Experimental section). It should nevertheless be noted that longer chains may exhibit a diminished propensity for multiple protonation. The presence of unprotonated amines has been empirically shown to accelerate the oligomerization rate of silicic acid. Through binding to silanols, unprotonated amines facilitate localized activation by deprotonation, thereby aiding in their nucleophilic attack on silicic acid molecules and consequently expediting oligomerization.^[^ ^66^, ^67^ ^]^

**Figure 5:**
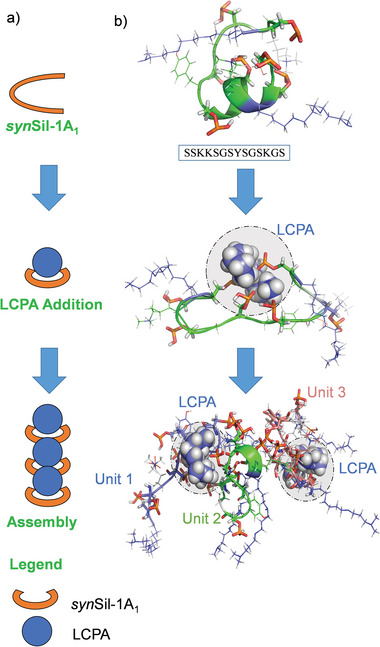
LCPA interaction and supramolecular architecture of *syn*Sil‐1A_1_ assemblies. a) Cartoon of tripeptide assembly, as observed in MD simulations. b) Snapshots of neat *syn*Sil‐1A_1_, *syn*Sil‐1A_1_ in the presence of LCPA, and four *syn*Sil‐1A_1_ copies in the presence of LCPA, each after 700 ns of an MD simulation. The figure vitalizes the binding of LCPA to the central region of *syn*Sil‐1A_1_ and the trimer formation. Statistical analyses of the MD data can be found in the Figure S7 (Supporting Information).

Further, note that in our experiments, ≈19% or ≈12% of the original signal intensities remained observable, respectively. The observed behavior much resembled that of R5 despite differing buffer composition and counterion type (see the Experimental section for details). Particularly, we again observed CSP along the entire primary sequence and similar CSP patterns for both probed concentrations (Figure 4c), suggesting that the self‐assemblies formed by *syn*Sil‐1A_1_, too, are based on repetitive objects with locally similar conformational repeats. Also, the signal intensities dropped significantly (and LCPA concentration‐dependent) upon self‐assembly (Figure S17, Supporting Information). The sizes of the objects again depended on the availability of LCPA counterions, which form the bridges between the peptide units. The most prominent CSP was observed for residue K_12_^*^, suggesting that the LCPA counterions primarily interact with this site (in agreement with the MD‐derived complexes; see **Figure** **5**). Again, a match between hydrodynamic radii derived for the self‐assembled structures from DOSY (= 0.7+/−0.1 and 1.6+/−0.1 nm for monomer and trimer) and MD simulations (+/−0.4) again corroborated the formation of tripeptide assemblies that serve as building blocks for larger oligomers as a threefold volume increasing upon self‐assembly in solution was observed. Figure 5 visualizes the MD‐derived assembly process for the three *syn*Sil‐1A_1_ units (see Figures S8 and S9, Supporting Information for details on the simulation and supplementary DOSY data). Also, in the case of *syn*Sil‐1A_1_, the trimers remained stable once formed in the simulations. However, it cannot be excluded that an equilibrium exists in the experiments with a small population of dimers.

Reduced dynamics of *syn*Sil‐1A_1_ upon self‐assembly further corroborated the resemblance of R5's behavior. This was evidenced by continuously increasing ^31^P‐*R*_2_ rate constants with growing LCPA availability (Figure S16, Supporting Information), confirming the continuous self‐assembly of more tripeptide units with increasing counterion concentration.

Hence, for both, R5 and *syn*Sil‐1A_1_, the combined NMR and MD data indicate that the solution‐state template structures are constituted of repetitive tripeptide building blocks that supra‐assemble into larger structures (reminiscent of fractal polymers).^[^ ^45^, ^58^, ^68^ ^]^ The size of the supra‐molecular assemblies thereby depends on the counterion availability.

### 2.2 Peptide Templated Silica Precipitation by Real‐Time NMR

In the next step, we followed the formation of silica templated by the R5 and *syn*Sil‐1A_1_ self‐assemblies. It should be noted that the presented data focuses on the evolution of the peptide component, but naturally, the silica component of the systems evolves as well. However, this latter perspective has already been studied extensively. By solid‐state NMR spectroscopy of ^29^Si under MAS conditions, exclusively Q^3^ and Q^4^ resonances have been reported.^[^ ^69^ ^]^ For the case presented herein, the conversion into these species takes off from a mixture of Q^0^ and Q^1^ silicate species (see the Figure S21, Supporting Information). Furthermore, during the silica formation process, the entire peptide was reported (under some conditions) to be in contact with the silica, which matches our observations well.^[^ ^38^ ^]^ At the same time, the N‐terminus of R5 has been reported to directly interact with the silica inner surface upon completion of the particle formation.^[^ ^38^, ^70^, ^71^ ^]^ This is also in line with our observation that the SKKS motifs are phosphate‐bound in the assemblies reported above.

#### 2.2.1 R5 Peptide

**Figure 6:**
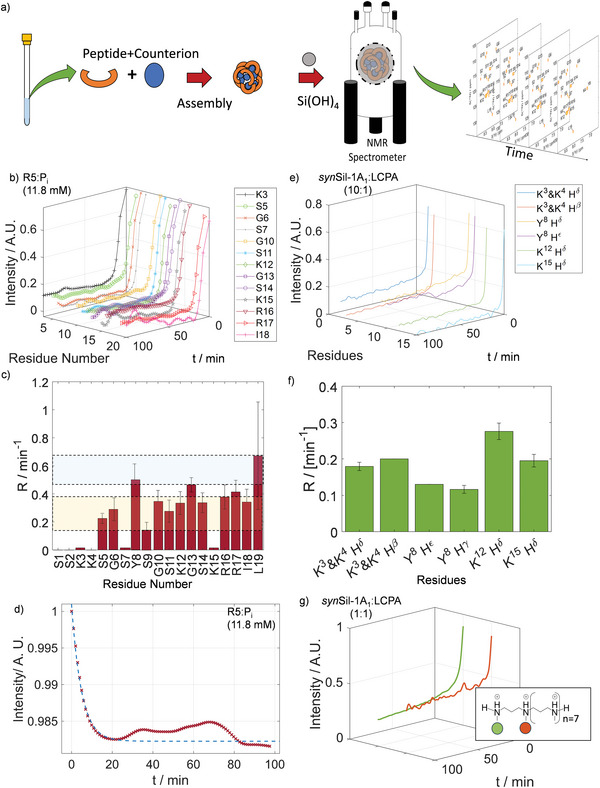
Real‐time monitoring of silica co‐precipitation. a) Scheme of the process for real‐time precipitation assays, shown exemplarily for *syn*Sil‐1A_1_ and LCPA. b) Time traces of signal intensities for the observable residues of R5 upon the addition of silicic acid. c) Decay rate constants for the residues of panel (b), obtained by fitting the data to exponential functions (see the Figures S22–S26, Supporting Information). The C‐terminal residue show the highest decay rate. The blue and yellow shades indicate faster and slower relaxing residues, respectively. d) Time traces of signal intensity of real‐time monitoring from the phosphate‐ion perspective by ^31^P NMR e) Time traces of signal intensities for the observable side‐chains of synSil‐1A_1_ upon addition of silicic acid. f) Signal decay rates for the function in panel (e). g) Time traces of signal intensity of real‐time monitoring from LCPA methyl groups.

For R5, we induced precipitation of silica by co‐dissolving 25 mm silicic acid (freshly generated from TMOS) and R5 in 11.8 or 50 mm aqueous phosphate solutions at pH 6.5 The precipitation event was monitored by ^1^H‐^15^N SOFAST HMQC (selective optimized flip angle short transient heteronuclear multi quantum coherence) NMR,^[^ ^72^ ^]^ yielding a 2D correlation spectrum every 2.5 min to detect those tripeptide units that remained in solution. NMR detection started immediately after mixing of the samples and insertion into the spectrometer (**Figure** **6**a). As a result, residue‐resolved real‐time monitoring could be achieved. Upon R5/P_i_/silica co‐precipitation, the NMR signals gradually disappeared as the silica precipitation proceeded. The time traces of the resulting normalized ^1^H‐^15^N cross‐peak signal amplitudes are shown in Figure 6b, together with signal decay rates describing the pace of signal loss (Figure 6c). After completion of the precipitation event, ≈17% of the peptides remained dissolved, based on the residual NMR signal intensities. Note that no changes in the NMR spectra and no visible precipitation were observed within one hour in the absence of any P_i_.

The signal decay indicates that the tripeptide complexes are in exchange with the large self‐assemblies. The precipitating assemblies then draw more and more free tripeptide units out of the solution while the material formation event proceeds. Hence, the process is monitored from the viewpoint of the “unbound” species.

Most strikingly, we observed varying decay rates for different R5 residues, indicating a process in which different residues are immobilized at different stages – contrary to classical co‐precipitation mechanisms for which a homogenous signal reduction would be expected.^[^ ^16^ ^]^ This observation matches our earlier work on silica‐precipitating peptides containing RRIL motifs.^[^ ^36^ ^]^ In such a process, the surface residues of the assemblies lose their signals at a rate different from that of the core residues. This observation was attributed to an initial silica nucleation on the templates’ surfaces, which immobilizes adjacent amino acids and suppresses their NMR signals. Subsequently, a buildup of silica‐peptide layers and precipitation of the entire silica/peptide assembly leads to complete signal loss. These findings are further well in line with previously described simple model systems of diatom bio‐silicification.^[^ ^14^, ^73^, ^74^, ^75^ ^]^

For the experiments reported herein, the C‐terminal R5 residues I18 and L19 lose their signal intensities faster than all other residues (Figure 6c). This observation can readily be explained by the mechanism described above. In such a scenario, the phosphate‐bound RRIL motifs are localized at the surface of the self‐assemblies. The fast exchange between free and bound tripeptide units entails an exchange‐averaged signal intensity, which is reduced fastest during the solidification process for residues close to the silica nucleation site at the solvent‐accessible surface of the assemblies.

Notably, the RRIL residues also showed the highest signal intensities in the HSQCs (Figure 2) of the trimers in solution before silicification, indicating that the solution‐state properties are, to some degree, retained during precipitation. Similar observations in the context of stimuli‐responsive polymers and biomimetic designer peptides match these findings.^[^ ^62^, ^76^, ^77^, ^78^, ^79^ ^]^

#### 2.2.2 *syn*Sil‐1A_1_

For the non‐isotope enriched *syn*Sil‐1A_1_, real‐time monitoring could only be achieved by a series of ^1^H NMR spectra of the peptide sidechains, as the ^1^H‐^31^P HSQC did not allow for sufficient time resolution. Although residue resolution could not be achieved due to overlap of the side chain signals of different residues, we again observed that different resonances decayed with different rates (Figure 6e,f), indicating again a complex two‐stage solidification mechanism as described above for R5. Upon completion of the precipitation event approx. 20% and 9% *syn*Sil‐1A_1_ remained dissolved at low and high molar ratios of LCPA, respectively. Again, no changes in the NMR spectra and no visible precipitation were observed within one hour in the absence of LCPA.

We further confirmed that the *syn*Sil‐1A_1_/LCPA, as well as R5/P_i_ self‐assemblies, retain intact peptide‐counterion complexes throughout the precipitation events by real‐time monitoring of the LCPA counterions during silicification by ^31^P and ^1^H detection, respectively (Figure 6d,g). Both counterions decayed at a rate that was similar to that of the R5 and *syn*Sil‐1A_1_ peptides upon silica exposure.

### 2.3 Self‐Assembly Size and Structure Determine Nanoparticle Morphology

The translation of the solution‐state assemblies’ properties into solid materials with controlled morphology was shown by SEM (scanning electron microscopy) of the resulting solids on the nm to µm scale and MD simulations of the self‐assembled templates.

**Figure 7:**
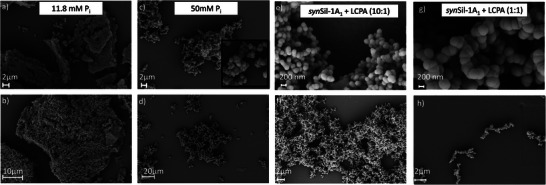
Scanning electron micrographs of the silica particles formed by R5/P_i_ – and *syn*Sil‐1A_1_/LCPA‐based assemblies. a,b) Porous, amorphous silica material yielded by precipitation with R5 in the presence of 11.8 mM P_i_. c,d) budded spherical silica particles, yielded by precipitation with R5 in the presence of 50 mM P_i_. e,f) spherical silica particles yielded by precipitation with synSil‐1A_1_/LCPA (10:1). g,h) spherical silica particles yielded by precipitation with synSil‐1A_1_/LCPA (1:1), resulting in larger spheres than for *syn*Sil‐1A_1_/LCPA (10:1).

**Figure** **7** shows electron micrographs for both systems for all probed conditions. For R5, at P_i_ concentrations of 11.8 mm, large porous silica “platelets” were formed, while at 50 mm, monodisperse budding spherical particles emerged with a diameter of ≈1 µm. For *syn*Sil‐1A_1_, monodisperse spherical particles emerged for both, high and low LCPA counterion concentrations, yet with significantly different sizes, changing from. 173+/‐26 nm to 238+/‐36 nm upon increasing the LCPA concentration (Figure 7e‐h; Figure S27, Supporting Information).

Note that the SEM images do not exclude the possibility that the observed structures are constituted by smaller spherical particles (Figures S28–S32, Supporting Information), even though not as strongly pronounced as in the 50 mm P_i_ case.

To rationalize these findings, we conducted MD simulations of the peptides in the crowded phase (Figures S33–S37, Supporting Information). Eight of the tripeptide building blocks shown in Figures 3 and 5, respectively (as identified by NMR and modeled by MD above), were densely yet randomly packed in a simulation box. The peptide packing density was based on the DOSY‐derived *R_h_* (Figures S8 and S9, Supporting Information). We used a peptide density that matched the density of the NMR‐identified trimers. The remaining void space was filled with explicit water, and the system was then evolved for >1 µs by all‐atom MD runs (for all details, see the Experimental section).

**Figure 8:**
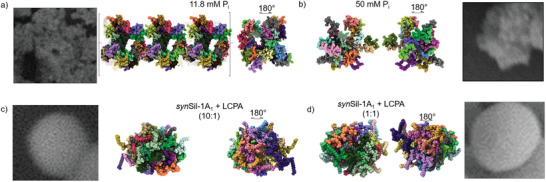
MD simulations of assemblies. a,b) Snapshot after 1000 µs of supramolecular R5:P_i_ assembly with 11.8 mm P_i_ and 50 mm P_i_. c,d) Snapshots after 1000 and 1000 µs, respectively, of supramolecular *syn*Sil‐1A_1_:LCPA assembly at 1:1 and 1:10 molar ratios. The EM insets highlight the qualitative match between simulated assemblies and the observed silica morphology. As the experimental and simulated assemblies exist on significantly different length scales, this morphological similarity is indicative of a scale‐free, i.e., fractal spatial organization of the assemblies. Note that R5 and 11.8 mm P_i_ led to structures that exceeded the simulation box size, as indicated by the square brackets. The different colors in the models indicate different peptide units. For the positioning of the RRIL motifs in panels a) and b), see the Supporting Information.

The resulting assembly structures and their comparison with the SEM images are shown in **Figure** **8**. (Our earlier studies clearly showed that the particles contain mainly silica.^[^ ^20^, ^29^ ^]^) For all four probed self‐assemblies, the simulated structures resemble, on a smaller scale, those observed by electron microscopy, from a porous network to budded particles to spheres. Hence, the silica coat templated by the peptide assemblies appears to be predetermined by the surface structure of the templates.

These observations align well with the mechanism independently derived from NMR above, silica nucleation on the peptide/counterion self‐assembly surface prior to complete coating. This mechanism leads to defined bioinorganic nanoparticles. As a result, the silica microstructures in Figures 7 and 8 display a highly conserved morphology (i.e., low size and shape dispersity) compared to purely inorganic silica (Figure S28, Supporting Information) with little to no deviation between the different particles.

Importantly, the length scale of the assemblies observed in our MD simulations (Figure 8) is approximately 100 times shorter than the equivalent length scale of the assemblies observed in SEM experiments. Thus, the morphological similarity between the two supports the possibility of a scale‐independent, i.e., fractal organization of the assemblies. Indeed, the match between experiment and simulation was quantitatively confirmed by the analysis of particle fractal dimensions (all details can be found in the Experimental section and Figure S33, Supporting Information). Further, it should be noted that the silica shell around the self‐assembled templates is typically several tens of nanometers thick, which can already partially account for the observed size differences.^[^ ^36^ ^]^

Specifically, the fractal dimension *d_f_* is a polymer physics‐based measure of scale‐invariant morphology. It can be extracted from MD trajectories using a framework described recently by Polyansky et al.^[^ ^55^ ^]^ We found that simulated and experimental *d_f_* values correlate quantitatively, which provides further support for the quality of the performed simulations as well as corroborates the idea of a fractal organization of the assemblies (Figure S33, Supporting Information). We found a close correlation in the relative ordering of *d_f_* values, with a small yet systematic underestimation of ≈20%. Interestingly, a peptide‐peptide contact (Table S3, Supporting Information) analysis showed that despite different morphologies and, hence, also *d_f_* values, the number of peptide‐peptide contacts within the self‐assemblies remained constant, independent of the counterion concentration. This finding, thus, again underlines the above deduction that the self‐assemblies consist of structure‐conservative peptide repeats, with tripeptide subunit assemblies conserved in structure.

The positioning of the P_i_ and LCPA counterions in the structures shown in Figure 8 are shown in Figure S37 (Supporting Information). We find that the phosphate ions are bound to the SKKS and RRIL motifs. For *syn*Sil‐1A_1_, a similar observation was made for R5. Also, we are now providing new data to show the positions of the LCPAs in the complexes. In particular, the phosphorylated serine 5 and 14, as well as all K* side‐chains, showed stable contacts with LCAP in the MD simulations.

It should furthermore be noted that the analysis of the fractal dimension shown in the Supporting Information is a quantitative measure derived from an average over the entire MD trajectories (Figures S34–S36, Supporting Information). While the images in Figure 8 only show representative snapshots for visualization purposes, the quantitative comparisons in Figure S33 (Supporting Information) are statistically validated. The presented data should be interpreted with this piece of information in mind.

## 3 Conclusions

In summary, we demonstrate how the integration of NMR and MD developments into existing methodological and experimental settings, as those often found in integrative structural biology, can aid the elucidation of the mechanism and precursor structures of biomimetic mineralization events at atomistic detail. As a result, the structure‐function relation of R5/P_i_ – and *syn*Sil‐1A_1_/LCPA‐based template systems could be revealed.

In particular, we could show that both, R5 and *syn*Sil‐1A_1_ self‐assemblies consist of tripeptide building blocks, which assemble into larger structures. The latter sizes and morphologies are dependent on the number of supramolecular ion bridges between the peptides and available counterions.

These template assemblies then catalyze silica nucleation on their surface, followed by complete surface‐coating.

**Figure 9:**
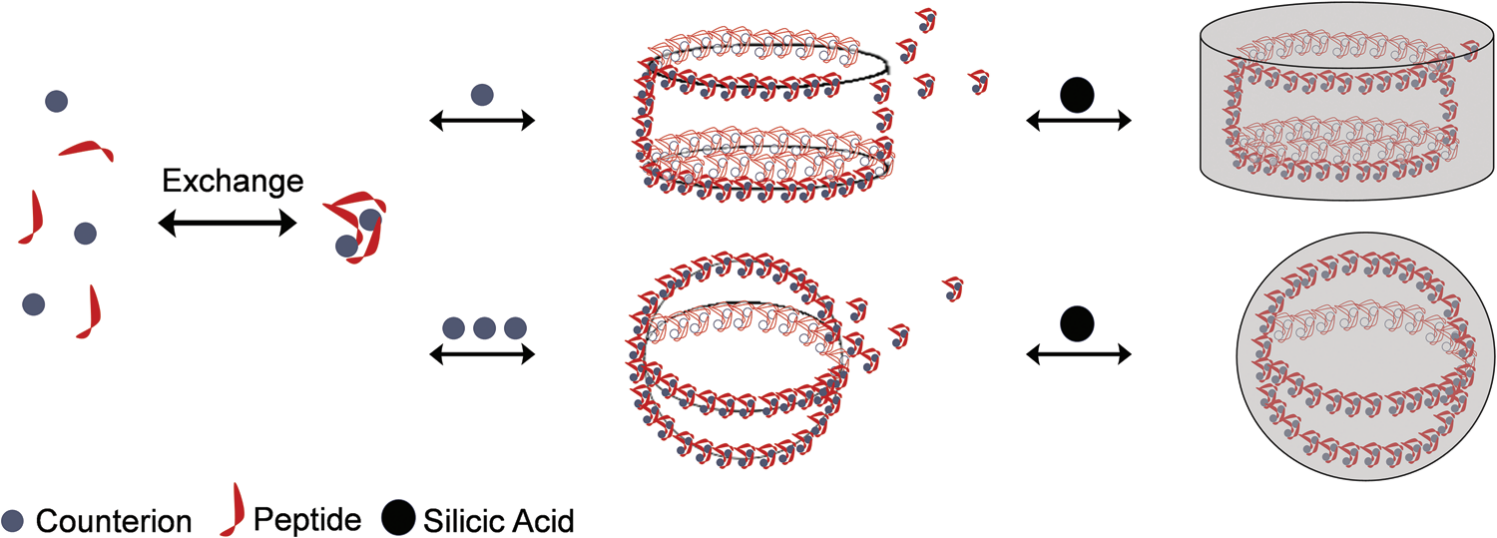
Schematic representation of peptide self‐assembly and coating in the in presence of counterions and later addition of silicic acid. Scheme not to scale.

Hence, the properties of the solute templates are maintained and effectively translated into solid nanoparticles. The derived mechanism is summarized in **Figure** **9**.

Our findings report for the first time an atomistic structure of self‐assembled peptide templates and follow these structures through the entire silification process to the solid state. In other words, most research on R5 and silaffins has focused on the structure of the solid‐state parts of these systems, but herein, we provide a detailed description of the solution state part and its translation into the solid state.

The reported findings feature further interesting implications: Recently, the formation of condensed liquid phases^[^ ^25^, ^80^, ^81^, ^82^ ^]^ has received ample attention as it was suggested that such processes are involved in the formation of cellular compartments without the need for membranes.^[^ ^83^ ^]^ The formation of large self‐assemblies, which yet remain in solution and display complex internal structural dynamics, resembles such a non‐classic phase separation. It might, therefore, be possible that peptide condensation in solution is not only involved in the formation of cellular compartments but might also play a role in biomineralization events by predefining the morphology of a solid required by a living organism. Indeed, to produce a specific material on demand, control over the necessary precipitation pathway needs to be established already at the reversible precursor stage.

## 4 Experimental Section

### Peptide Expression

R5 was subcloned as a His‐tagged SUMO‐fusion construct into a pET‐21a(+) expression vector and transformed into E. coli Rosetta2 cells. For protein expression, bacteria were grown at 37 °C in LB media until transferred to M9 for ^13^C and/or ^15^N labeling (^13^C_6_ glucose and/or ^15^N ammonium chloride added at 1 g L^−1^). Cells were induced with isopropyl‐β‐d‐thiogalactopyranoside at an optical density corresponding to A600 = 0.6 and incubated at 30° C overnight. Cells were homogenized in a solution of TRIS (25 mm), NaCl (100 mm), and β‐mercaptoethanol (2 mm) at pH 8. The resulting supernatant was purified by Ni^2+^‐affinity chromatography, and fractions pooled when a mass of 15.470 kDa (^15^N labeled) and 16.449 kDa (^13^C/^15^N labeled), were found in LC‐MS analyses. Cleavage of His and SUMO (small ubiquitin‐related modifier)‐tag was achieved by SUMO protease digestion overnight, confirmation was by mass spectrometry, reporting masses of 13.448 kDa of His‐SUMO‐tag (^15^N labeled), and 14.355 kDa of His‐SUMO‐tag (^13^C/^15^N labeled).

The cleaved peptide mixture was purified by Kromasyl C4 semi‐preparative RP‐HPLC column on a Waters Prep 150 System using a gradient from 5% – 65% of water/acetonitrile (0.08% v/v) in water/trifluoroacetic acid (0.01% v/v) over 30 min at a flow rate of 5 mL min^−1^. The fractions with UV absorption above 70 mAu were collected automatically. For analysis, 15 µL of each fraction was directly injected into a Thermo Fisher HPLC‐MS system to identify product‐containing fractions. Fractions were finally pooled accordingly and lyophilized.

The *syn*Sil‐1A_1_ was produced as detailed in the earlier publication. All details can be found therein.^[^ ^20^ ^]^

### NMR Spectroscopy

All spectra were acquired at 25 °C on a Bruker NEO 600 MHz spectrometer, equipped with a cryogenically cooled Prodigy TCI probe head, except for ^31^P‐ and ^29^Si‐detected spectra, which were recorded on a Bruker NEO 500 MHz spectrometer, equipped with a BBFO Prodigy cryogenic probe head. All pulse sequences were taken from the Bruker TopSpin 4 library except for the ^31^P‐*R*_2_ experiments outlined in the Supporting Information.

Assignment of the resonance type of *syn*Sil‐1A_1_ was achieved by a combination of TOCSY (total correlation spectroscopy), selective COSY (correlation spectroscopy), and NOESY (nuclear Overhauser spectroscopy), acquired in States‐TPPI mode for quadrature detection, and using “mlevgpphw5”, “cosygpphppw5” and “noesygpph19” pulse sequences. TOCSY spectra were recorded with a spectral width of 8196.721 Hz in both dimensions and 16 scans. COSY spectra were recorded with a spectral width of 7812.500 Hz in both dimensions with 32 scans, NOESY spectra with a spectral width of 5882.353 Hz in both dimensions with 16 scans and mixing times of 500 and 200 ms, respectively. The signal assignment of the R5 backbone resonances was achieved by a combination of HNCO, HNCACB, HNN, and HN(C)N spectra (see Table S1, Supporting Information for the resonance assignment). Carrier frequencies were chosen as ^1^H 4.7 ppm, ^15^N 117.0 ppm, and ^13^C 101.0 ppm.

HSQC spectra were recorded with the Bruker pulse sequence “hsqcetf3gpsi” with a spectral width of 2128.799 Hz in F1 and 9615.385 Hz in F2, using 32 scans and in‐plane echo‐antiecho detection. SOFAST‐HMQC^[^ ^72^, ^84^, ^85^ ^]^ were recorded by the “sfhmqc3gpph” pulse program in States‐TPPI mode for QUADRATURE detection with the same spectral width settings as in the recorded HSQC. 8 scans were chosen 8 for real‐time precipitation assays and 32 for other purposes. HCON^[^ ^86^ ^]^ experiments were recorded with a spectral width of 1824.688 Hz in F1 and 7419.52 Hz in F2 dimension and 64 scans IPAP detection mode.

^1^H‐DOSY spectra were recorded by the “stebpgp1s19” pulse program with 128 data points in a linear variation of z gradient strength from 0 to 0.1 T^2^m^−1^. The diffusion delay was 60 ms or 20 ms. Data were analyzed using GNAT,^[^ ^87^ ^]^ by integrating significant peaks of the sidechain region and fitting the integral versus gradient strength curve to the Stejskal‐Tanner equation.^[^ ^88^ ^]^ Hydrodynamic radii were extracted by the Stokes‐Einstein law, assuming a spherical model.^[^ ^89^, ^90^, ^91^ ^] 31^P DOSY were recorded by “dstebpgp3s” pulse program with 40 points in a linear variation of z gradient strength from 0 to 0.1 T^2^m^−1^. The diffusion delay was 100 ms.

HNCO, HNN, and HNCACB were recorded by pulse programs “b_hncogp3d”, “best_hnngpwg3d”, and “hncacbgpwg3d”. HNCO was recorded with spectral width and offset frequencies of 8196.721 Hz and 4.7 ppm for ^1^H, 2128.799 Hz, and 117.0 ppm for ^15^N, and 2113.182 Hz, and 173.500 ppm for ^13^C. HNN^[^ ^92^ ^]^ was recorded with spectral widths and offsets of 6250.000 Hz and 4.669 ppm for 1H and 1824.689 Hz and 122.000 ppm for ^15^N. HNCACB was recorded with spectral width and offsets of 8196.721 Hz and 4.7 ppm for ^1^H, 2128.799 Hz, and 117.0 ppm for ^15^N, and 12 073.750 Hz and 43 ppm for ^13^C.

^15^N relaxation rates were recorded by ^1^H‐^15^N correlation spectra with the pulse program “hsqct2etf3gpsi3d” with spectral widths and offset frequencies of 9615.385 Hz, and 4.7 ppm for ^1^H, 2128.799 Hz, and 117.0 ppm for ^15^N and 2113.182 Hz.

All spectra were analyzed using Topspin 4.1 and Topspin 4.2 as well as MATLAB R2020a. NMRpipe^[^ ^93^ ^]^ and SPARKY^[^ ^94^ ^]^ were used to process all acquired 3D Data (HNCACB, HNN, HNCO, *R*_2_). Data were zero‐filled to twice the number of points and apodized using a 60° shifted sine bell function prior to Fourier transformation. Baseline correction was achieved by a polynomial function in the frequency space.

Chemical shift perturbations were computed as

(1)

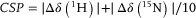

for ^1^H‐^15^N correlation spectra and as

(2)

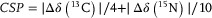

for ^13^C‐^15^N correlation spectra.

### Precipitation Assays

*R5*: 1 mg of the purified peptide was dissolved in K_2_HPO_4_/ KH_2_PO_4_ buffer (500 µL, either 11.8 mm or 50 mm at pH 6.5, each containing 10% D_2_O to allow for analysis by NMR spectroscopy) and equilibrated for 24 h in the NMR tube prior to precipitation. Silicic acid was prepared by hydrolysis of tetramethylorthosilane (TMOS, 37 µL) in aqueous HCl (963 µL, 1 mm) followed by mixing and incubation for 10 min. 50 µL of the resulting solution was then added to the protein sample, yielding a concentration of Si(OH)_4_ (25 mm).

Then the homogeneously labeled ^15^N R5 sample was tracked by recording SOFAST‐HMQC (sfhmqcf3gpph) spectra every 2 min and 20 s over the course of 7 h, using a Bruker NEO 600 spectrometer equipped with a cryogenically cooled TCI probe head. Spectra were acquired in States‐TPPI mode for quadrature detection with carrier frequencies centered at 4.7 ppm and 117.0 ppm for ^1^H and ^15^N, respectively.

Counterion ^31^P detected real‐time assay was traced by recording 1D spectra every 59 s over the course of 15 h using a Bruker NEO 500 MHz spectrometer equipped with a BBFO Prodigy cryogenic probe head with 8 scans.

The “high” counterion cases were chosen as those that worked most reliably according to the available literature.^[^ ^95^ ^]^ The “low” concentration cases were then chosen according to ref.,^[^ ^20^, ^58^ ^]^ where it was shown that under such conditions (which approach biomimetic ones), silica formation was still taking place, yet the morphologies differed from those found for the higher concentrations.

*synSil‐1A_1_*: synSil‐1A_1_ and LCPA were dissolved in Tris buffer (25 mm) at pH 7.5 to equimolar conditions at 1 mm concentrations in 90% H_2_O/ 10% D_2_O. These conditions match those used in earlier studies of synSil‐1A_1_.^[^ ^20^ ^]^ Otherwise, the process was identical to the method described for R5, except for tracking the intensity change via 1D proton‐only spectra, recorded every 2 s with a spectral width of 9615.385 Hz and frequency offset of 4.695 ppm and binomial water suppression.

### Scanning Electron Microscopy

Precipitates were centrifuged, separated, and subsequently washed three times with H_2_O (1 mL). The pellet was resuspended in H_2_O (1 mL) and diluted 1:10 with H_2_O. The resulting suspension (10 µL) was brought onto a ThermanoxTM coverslip and air‐dried. A layer of gold was added to all samples by sputter coating under high vacuum (Bal‐Tec SCD 005), and SEM imaging was then performed with a Zeiss SEM supra 55 VP at 20 kV. The images were evaluated using the ImageJ software package. The reported radii are those found in the aggregated particles. To ascertain significance, only if two opposite edges of the particle circumference could be observed, the radius was evaluated (see the Supporting Information).

### Molecular Dynamics Simulations

Monomer simulations at different counterion concentrations were carried out in 4.65×4.65×4.65 nm^3^ (neat R5), 6.37×6.37×6.37 nm^3^ (R5:11.8 mm Pi), 3.15×3.15×3.15 nm^3^ (R5:50 mm Pi) and 4.28×4.28×4.28 nm^3^ (*syn*Sil‐1A_1_) water boxes for > 700 ns length. Each simulation was repeated three times. Simulations of sub‐assemblies were carried out in 6.386×6.386×6.386 nm^3^ (4R5:11.8 mm Pi), 6.36×6.36×6.36 nm^3^ (4R5:50 mm Pi) and 3.94×3.94×3.94 nm^3^ (*syn*Sil‐1A_1_:LCPA, 3:1), 4.88×4.88×4.88 nm^3^ (*syn*Sil‐1A_1_, 1:1) water boxes for >700 ns, respectively. Simulations with 25 copies were carried out in 7.26×7.26×7.26 nm^3^ (R5:11.8 mm Pi), 71.5×7.15×7.15 nm^3^ (R5:50 mm Pi) and 5.20×5.20×5.20 nm^3^ (*syn*Sil‐1A_1_:LCPA, 3:1) and 5.70×5.70×5.70 nm^3^ (*syn*Sil‐1A_1_:LCPA, 1:1) water boxes for >1 µs (again three replica were computed). See Tables S2 and S3 (Supporting Information) for further details on the simulated systems. Starting structures were yielded by the PEPstrMOD^[^ ^96^, ^97^ ^]^ server for peptide structure prediction. For the trimer simulations, four copies of the peptide structure generated by the last snapshot of the monomer simulation were placed in a box randomly. For simulations of the peptide assemblies, 25 copies of the structures yielded by the trimer simulations were placed in a box with the density matching those found in the DOSY NMR experiments (see below). Trajectories were calculated with the YASARA software package^[^ ^98^, ^99^ ^]^ using the AMBER14N^[^ ^100^ ^]^ force field. The conditions were chosen mirroring the experimental concentrations, pH, and temperatures of the NMR experiments. All systems were simulated in explicit water using a three‐point TIP3P^[^ ^101^ ^]^ water model. Sidechain protonation was considered using the routines built into the YASARA program package. The routine computed full protonation of all side chains, in agreement with the documented pKa values arginine and lysine, as outlined in the main text. In contrast, phosphoserines were singly protonated. The final mass fraction of NaCl was adjusted to 1%. The systems were energy‐minimized and equilibrated with steepest descent minimization and simulated annealing in 500 steps with all atoms except water restrained and then again unrestrained for 500 steps of 2 fs. The production runs were carried out in 2 fs time steps, and snapshots were taken every 10 ps.

The cut‐off for nonbonded Coulombic interactions was at 10.5 Å in all three directions of space and treated by the smoothed particle Ewald method.^[^ ^102^ ^]^ MD simulations were carried out using 3D periodic boundary conditions in the isothermal‐isobaric (NPT) ensemble with an isotropic pressure of 1.013 bar and a constant temperature of 310 K. The temperature and pressure were controlled by Berendsen thermostat^[^ ^103^ ^]^ and “densostat” method by dynamic box size rescaling to maintain a density of 0.997gmL^−1^.^[^ ^99^ ^]^

For comparison with the NMR experiments, the radius of gyration was converted to *R_h_* by a factor of 1.1 according to refs.^[^ ^68^, ^104^ ^]^

(3) $$R_{g}/\;R_{h}=1.1$$

For MD simulations of R5 assemblies in the crowded phase, 25 copies of the tripeptide cluster were simulated for R5 in a cubic simulation box with 7.11 and 7.24 nm edge lengths. This yielded a density of *d* = 0.6194 g mL^−1^, which matched the density *d* derived from hydrodynamic radii found in the DOSY experiments (Figures S7 and S8, Supporting Information), *i.e*., such that *d* = 9⋅*m*(peptide)/4π*R_h_* ^3^, with *m*(R5) being the molecular weight of R5. The rest of the box was filled with explicit water. The peptides/counterion clusters were then let to self‐assemble until a stable plateau in terms of R_h_ was reached and then simulated for another 200 ns. Within ≈1000 ns, the assemblies formed spontaneously and then remained stable for the rest of the MD run. The last 200 ns were chosen for the calculations of the fractal dimensions (see Supporting Information). The simulation results were reproduced in three independent runs for both probed conditions. The same procedure was then repeated for *syn*Sil‐1A_1_ (at 5.06 and 5.20 nm edge length and a density of 0.8899 g mL^−1^, respectively). The fractal dimension (*d_f_)* was calculated by Equ (4), taking into account the calculated R_g,_ and valency (i.e., the number of peptide‐peptide contacts) as defined per monomer. As this is a deviation from the workflow in Polyansky et al.,^[^ ^55^ ^]^ this might contribute to the systematic deviation of calculated (*d_f_)*.

(4) $$\varphi \;=\frac{V_{mol}}{\frac{4}{3}\pi R_{g}^{3}}\;$$

(5) $$\;V_{mol}=\;\kappa *M_{w}$$

(6) $$\;d_{f}=\frac{3}{1-\frac{log\varphi }{log\left(valency\right)}}\;$$

φ: Compactness

V_mol_: Molecular Volume

κ: Prefactor by Polyansky et al.^[^ ^55^ ^]^ (= 1.21)

*d_f_* : Fractal Dimension

### Small Angle X‐Ray Scattering

SAXS measurements were performed by preparing solutions, filling them into glass capillaries with 1.5 mm diameter and 10 µm wall thickness (from Hilgenberg, Germany), sealing the capillaries vacuum‐tight, and measuring each capillary for 3 hours. X‐ray patterns were recorded using a microfocus X‐ray source with a copper target equipped with a pinhole camera (Nanostar, Bruker AXS) and a 2D position‐sensitive detector (Vantec 2000). All two‐dimensional SAXS patterns were radially averaged and background subtracted (background from a capillary with pure water for R5, background from water, and the respective amount of phosphate for R5+12 mm and R5+50 mm phosphate) to obtain the scattering intensities in dependence on the scattering vector q = 4π/λ sin(*θ*), with 2*θ* being the scattering angle and *λ* = 0.1542 nm the X‐ray wavelength. Without the capillary scattering, the background from water, as well as from phosphate dispersed in water, exhibits typical fluid scattering without any structure, i.e., constant scattering intensity, which shows that the phosphate is completely dissolved.

Data were fitted with the unified scattering function from Beaucage,^[^ ^105^, ^106^ ^]^ from which the radius of gyration R_g_ is obtained. For R5, additionally, the data from the protein data bank^[^ ^107^ ^]^ were used to calculate the scattering pattern using the software.^[^ ^105^ ^]^

### Statistical Analysis

For the data presented in Figure S13 (Supporting Information), the statistical significance analysis was performed via the Wilcoxon test. Pre‐processing: the data were normalized to yield a total probability density of 1. No outliers were excluded. All determined distances were directly fed into the statistical analysis. Data presentation: Size distributions as well as mean values and standard deviations. Sample size: 1000 radii for both peptides. Statistical methods used: Wilcoxon test. All p‐values were below 0.05. Used software: MATLAB 2023a.

## Conflict of Interest

The authors declare no conflict of interest.

## Supporting information

Supporting Information

## Data Availability

The data that support the findings of this study are available from the corresponding author upon reasonable request.
