# Supplementary material for: An Atomistic View on the Mechanism of Diatom Peptide‐Guided Biomimetic Silica Formation
Source: Adv Sci (Weinh). 2024 Jun 14;11(30):2401239. doi: 10.1002/advs.202401239 (PMC11321707; doi:10.1002/advs.202401239)
Supplement: Supplementary file 1 — Supporting Information [file ADVS-11-2401239-s001.pdf]

ADVANCED SCIENCE
Open Access

Supporting Information

for *Adv. Sci.*, DOI 10.1002/advs.202401239

An Atomistic View on the Mechanism of Diatom Peptide-Guided Biomimetic Silica Formation

Fanny Kozak, Dörte Brandis, Christopher Pötzl, Ludovica M. Epasto, Daniela Reichinger, Dominik Obrist, Herwig Peterlik, Anton Polyansky, Bojan Zagrovic, Fabian Daus, Armin Geyer, Christian FW Becker and Dennis Kurzbach**

Supporting Information

An Atomistic View on the Mechanism of Diatom Peptide-guided Biomimetic Silica Formation

Fanny Kozak, Dörte Brandis, Christopher Pötzl, Ludovica M. Epasto, Daniela Reichinger, Dominik Obrist, Herwig Peterlik, Anton Polyansky, Bojan Zagrovic, Fabian Daus, Armin Geyer, Christian FW Becker, Dennis Kurzbach**

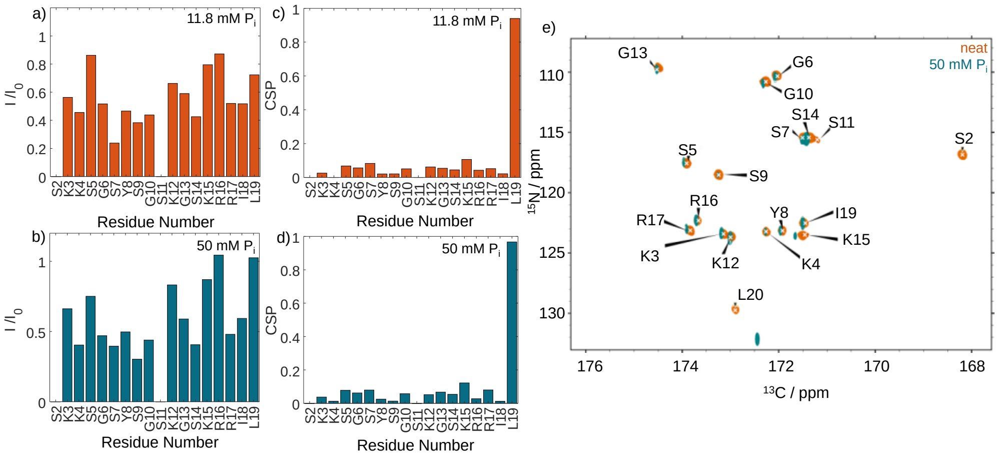

Figure S1. ^{13}C-detected NMR of R5. a,b) Intensity ratios (I/I_0) between resonances in the presence (I) and absence (I_0) of 12 or 50 mM P_i. obtained via $^{1}H^{N}$-CON NMR. c,d) CSP upon P_i exposure at concentrations of 11.8 and 50 mM, respectively. e) Comparison of $^{1}H^{N}$-CON spectra in the absence and presence of P_i (shown for 50 mM Pi, the spectrum at 11.8 mM looked identical).

To probe the structures of R5 within the assemblies by residue-resolved NMR, we employed an approach based on ^{13}C-^{15}N correlation spectroscopy. We employed the $^{1}H^{N}$-CON pulse sequence developed by Felli et al.[1] This detection scheme is much less prone to signal loss due to high molecular weights and slow molecular tumbling[2]. Figure S1a-b shows that the signals were reduced upon self-assembly, yet not as strongly (<40% at 11.8 mM P_i; <60% at 50 mM P_i) as in the ^{1}H-^{15}N HSQC shown in the main text, indicating that resonances of peptide units even within larger structures, which remain elusive in the HSQC, were traceable by ^{13}C-direct detection. The continuing signal amplitude reduction with increasing P_i concentration points towards continuously rising numbers of peptides trapped in the assemblies due to a higher number of P_i-salt bridges. Capitalizing on these experiments, the ^{13}C-^{15}N CSP upon self-assembly was again determined and found to be similar for both probed P_i concentrations (Figure S1), confirming (i) that the average conformational adaptions of R5 are independent of

the assembly size and (ii) that the structural adaptions within the assemblies are also P_i concentration independent.

Most strikingly, the ^{15}N transverse relaxation rate constants R_2 increased significantly when going from 11.8 to 50 mM phosphate when measured *via* ^{13}C-direct detection (Fig. S10). This clearly shows that the R5 self-assemblies continue to grow with increasing counterion concentrations and that the ^{13}C-detected experiments pick up an average between free species peptide trimers bound within the self-assemblies.

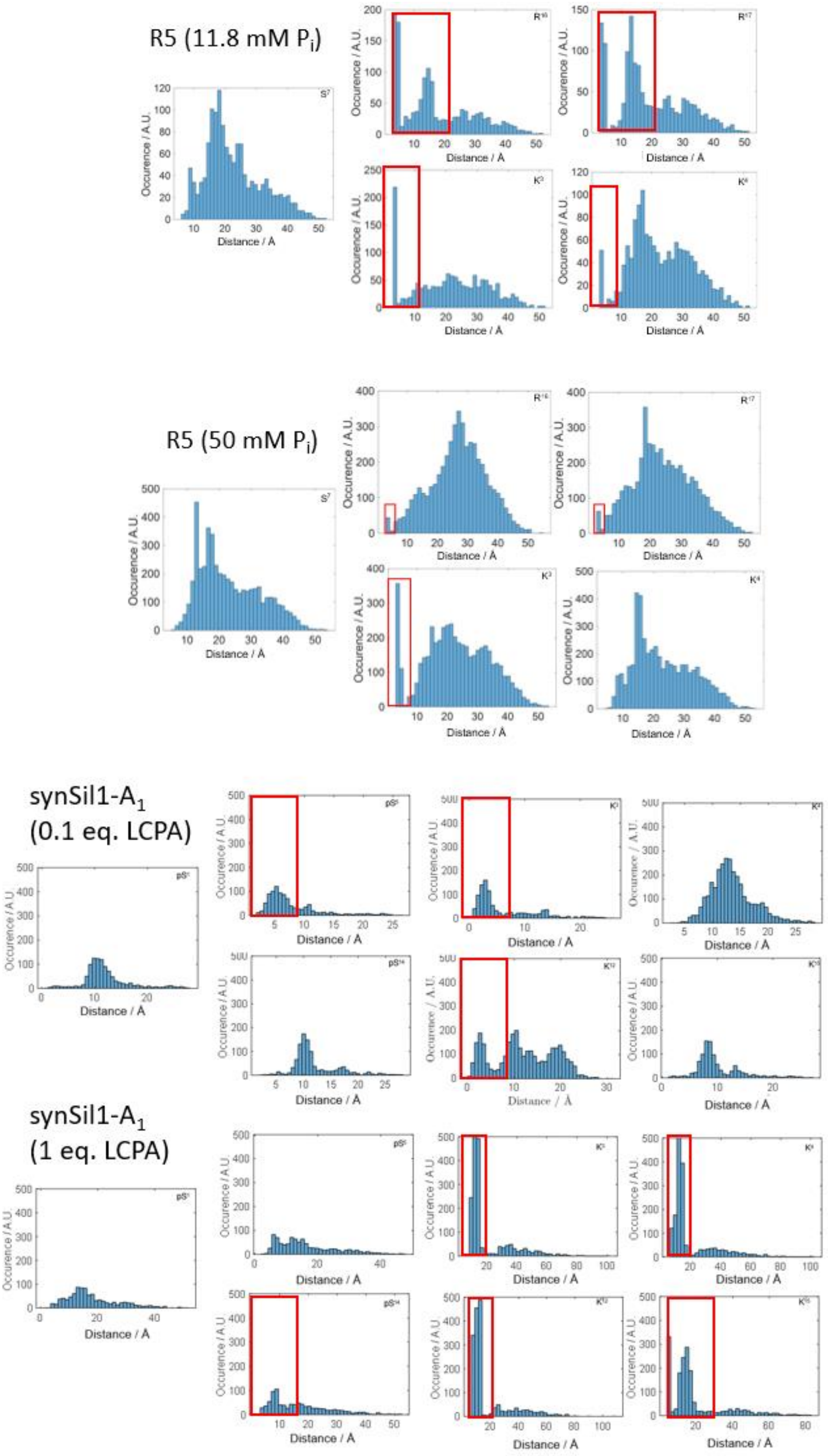

Figure S2: P$_i$-to-residue distance histograms for the R5 trimer at 11.8 mM and 50 mM P$_i$ (top) and of LCPA to *syn*Sil1-A$_1$ trimers (bottom) extracted from the MD simulations simulation. The distinct distance populations < 10 *Å* correspond to direct contact between a counterion and the residue indicated in the top right of each panel. The red marked peaks are clear signs of

short distances due to the coordination of the counterions to the sidechains of the respective residues.

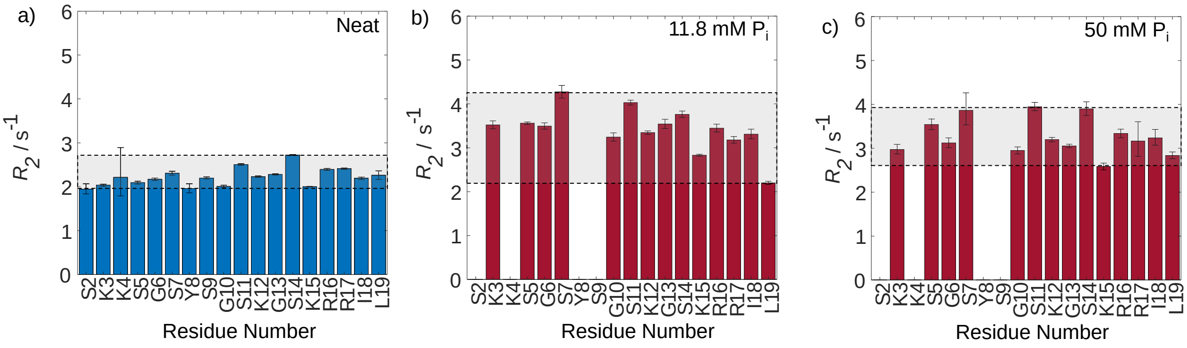

Figure S3. ^{15}N-R_2 rates of neat R5 (blue) and in the presence of 11.8 and 50 mM P_i (red). The grey shades indicate the range of observed values.

A particular behavior was observed for the R5 transverse relaxation rates ^{15}N -R_2. Higher values were observed along the entire primary sequence upon exposure to phosphate, indicating globally reduced nanosecond dynamics and/or chemical exchange effects. However, the relaxation rates only slightly changed with growing P_i concentration, and the chemical shifts remained unchanged while signal intensities significantly dropped (Fig. 2 of the main text).[3] This points towards the detection of free trimers in solution upon phosphate exposure.

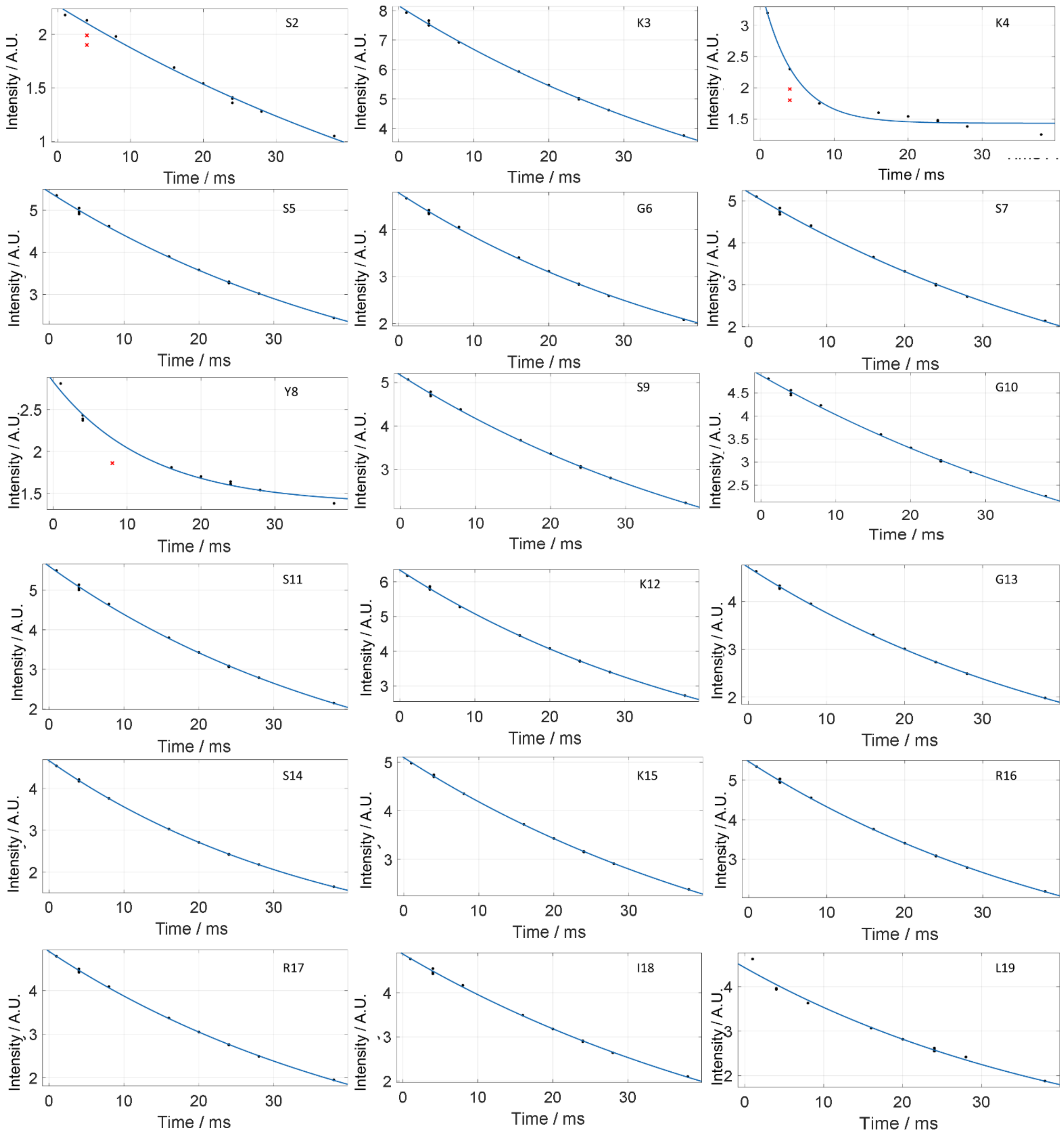

Figure S4. ^{1}H-^{15}N T_2 Relaxation Fits of R5 in water.

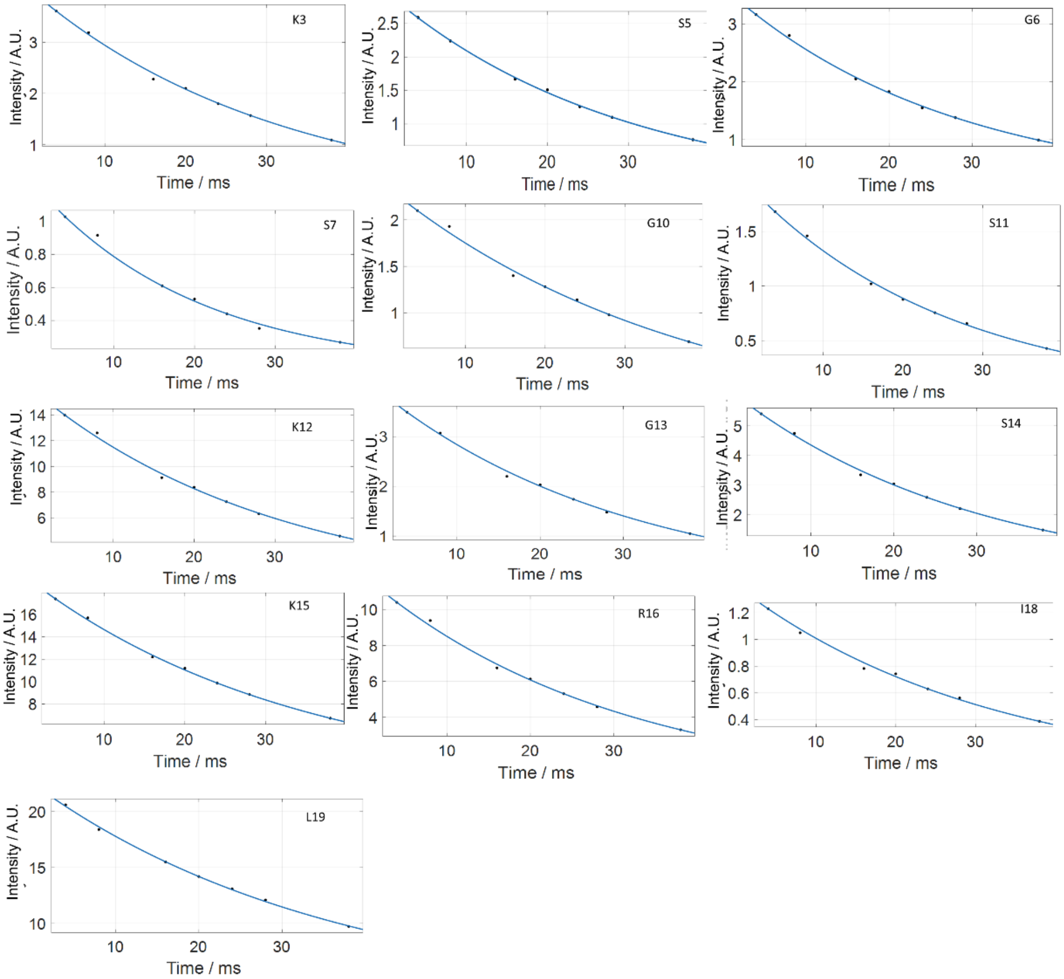

Figure S5. ^{1}H-^{15}N T_2 relaxation fits of R5 in 11.8 mM P_i. Residues not shown are broadened beyond detection.

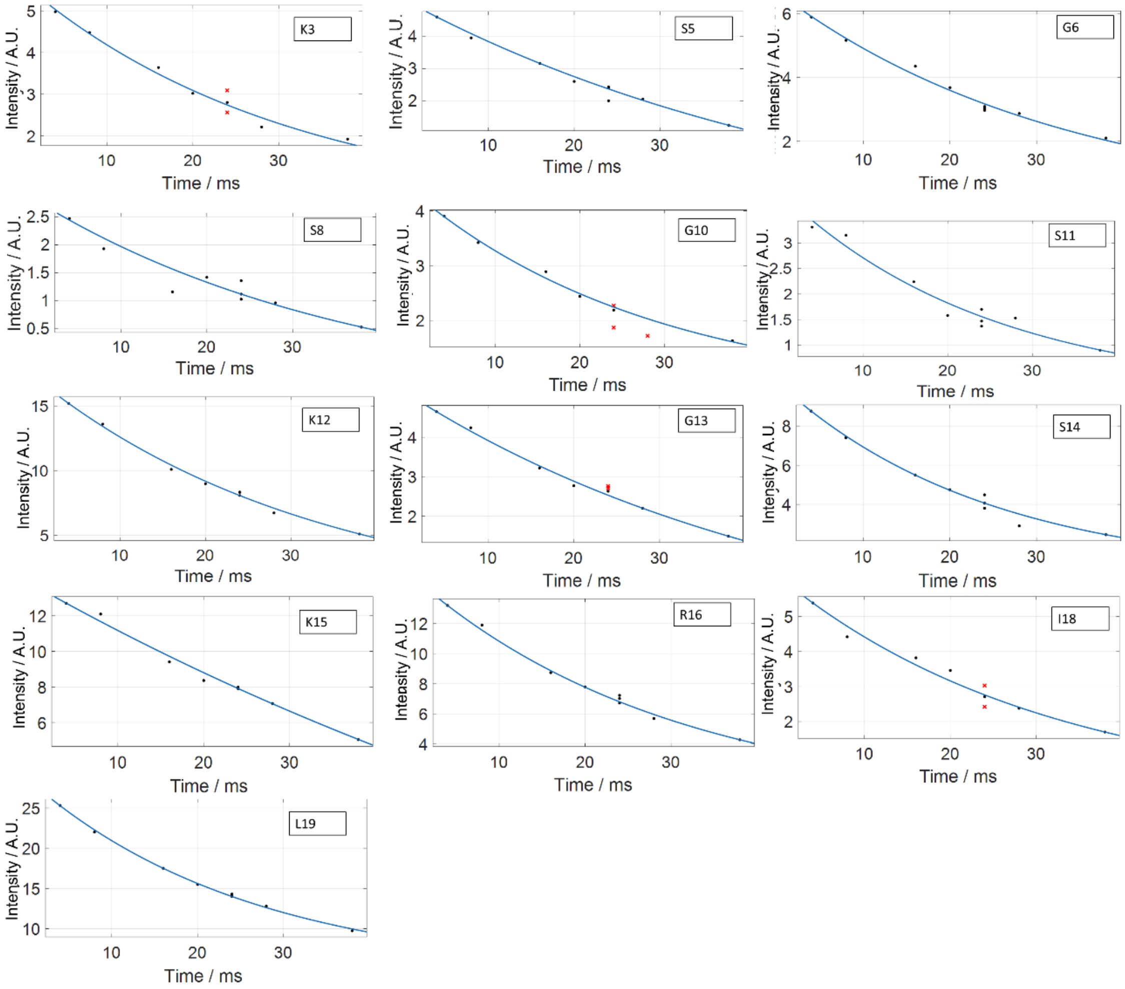

Figure S6. ^{1}H-^{15}N T_2 relaxation fits of R5 in 50 mM P_i. Residues not shown are broadened beyond detection.

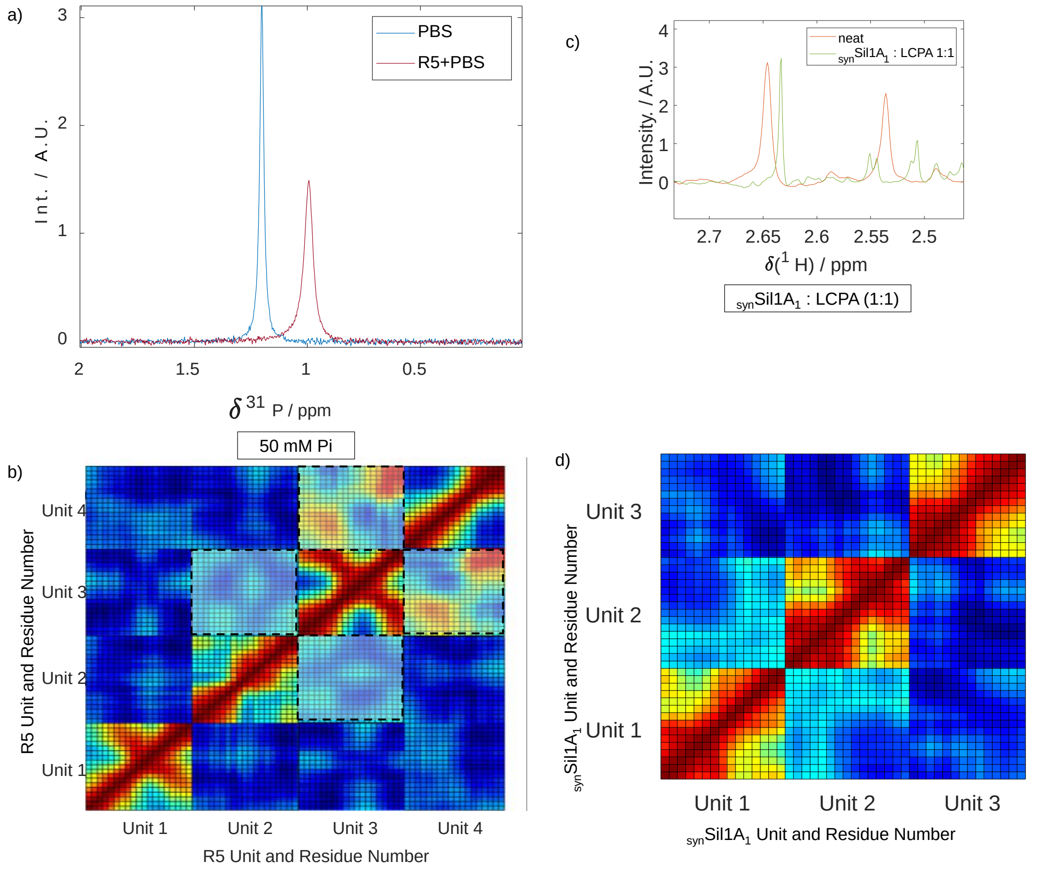

Figure S7. a) ^{31}P NMR of PBS and R5 in PBS at constant ionic strengths. b) Dynamic cross-correlation matrix from MD simulation of R5 in 50 mM P_i. c) ^{1}H NMR of 1:1 synSil-1A_1 : LCPA and LCPA in buffer. e) Dynamic cross-correlation matrix from MD simulation of 1:1 *syn*Sil-1A_1 : LCPA.

We found that the ^{31}P phosphate chemical shift of the phosphate ions changed significantly and that the linewidth increased upon exposure to R5, indicating close contact with the peptide (Figure S7a). Thus, the phosphate ions can act as “bridges” between the R5 peptides.

Dynamic cross-correlation matrices (DCCM), obtained by correlating the Cα-Hα vectors for each residue and snapshot of the MD trajectory (after plateauing, see below), then confirmed the ion bridge sites and the peptide trimer structure (Figure S7b). The DCCM correlate residues with similar motions, i.e., those within a stable structural motif (Fig. S7b); hence, mutual binding residues are strongly correlated. The DCCM confirmed firstly that three R5 peptides assembled, while one peptide remained uncorrelated. Secondly, positive matrix elements in the DCCM between the C-terminal RRIL confirm the phosphate binding site. Another N-terminal SKKS motifs acts as a secondary linker between the three units. This was observed for both probed P_i concentrations in 6 independent replica runs.

A similar trend was observed for synSil-1A_1. Similarly, to the changes in linewidth in ^{31}P Spectroscopy for R5, peaks assigned to LCPA in ^{1}H spectra support a direct binding, as derived

from CSP and line broadening observations upon self-assembly. (Figure S7c). The DCCM analysis of the MD simulations of the underlying peptide-LCPA complexes showed that, again, three synSil-$1A_1$ units exhibit correlated behavior (Figure S7d). We found LCPA forming contact sides, particularly with residues $pHyl^{12}$ and $pSer^9$.

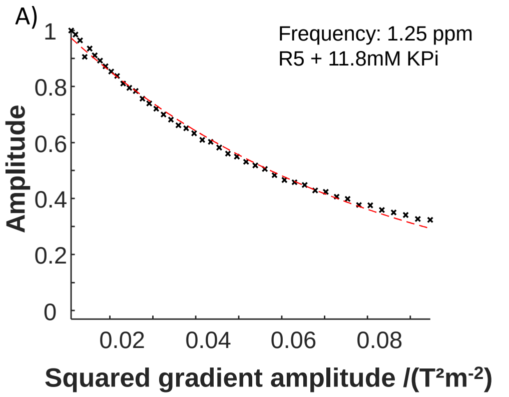

	D [10⁻¹⁰m²s⁻¹]	*r* ₕ[nm]
R5	3.53± 0.04	0.695± 0.08
11.8mM PBS	1.86± 0.04	1.320± 0.03
50mM KPi	3.22± 1.43	0.762± 0.234

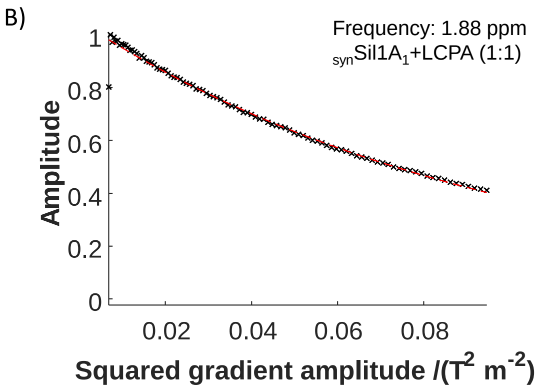

	D [10⁻¹⁰m²s⁻¹]	*r* ₕ[nm]
$_{syn}$Sil1A_1	3.12± 0.09	0.786± 0.023
+LCPA (10:1)	1.50± 0.57	1.640± 0.10
+LCPA (1:1)	3.47± 0.26	0.707± 0.050

Figure S8. A & B) DOSY data for R5 and *syn*Sil-$1A_1$ at different counterion concentrations. At low counterion concentrations, a doubled R_h was found relative to the free peptides in accordance with earlier reports.[4] The resulting three-fold increase in volume suggests three peptide units per cluster. It should be noted that the hydrodynamic radii found by DOSY correspond very well with the radii found in the MD simulations.

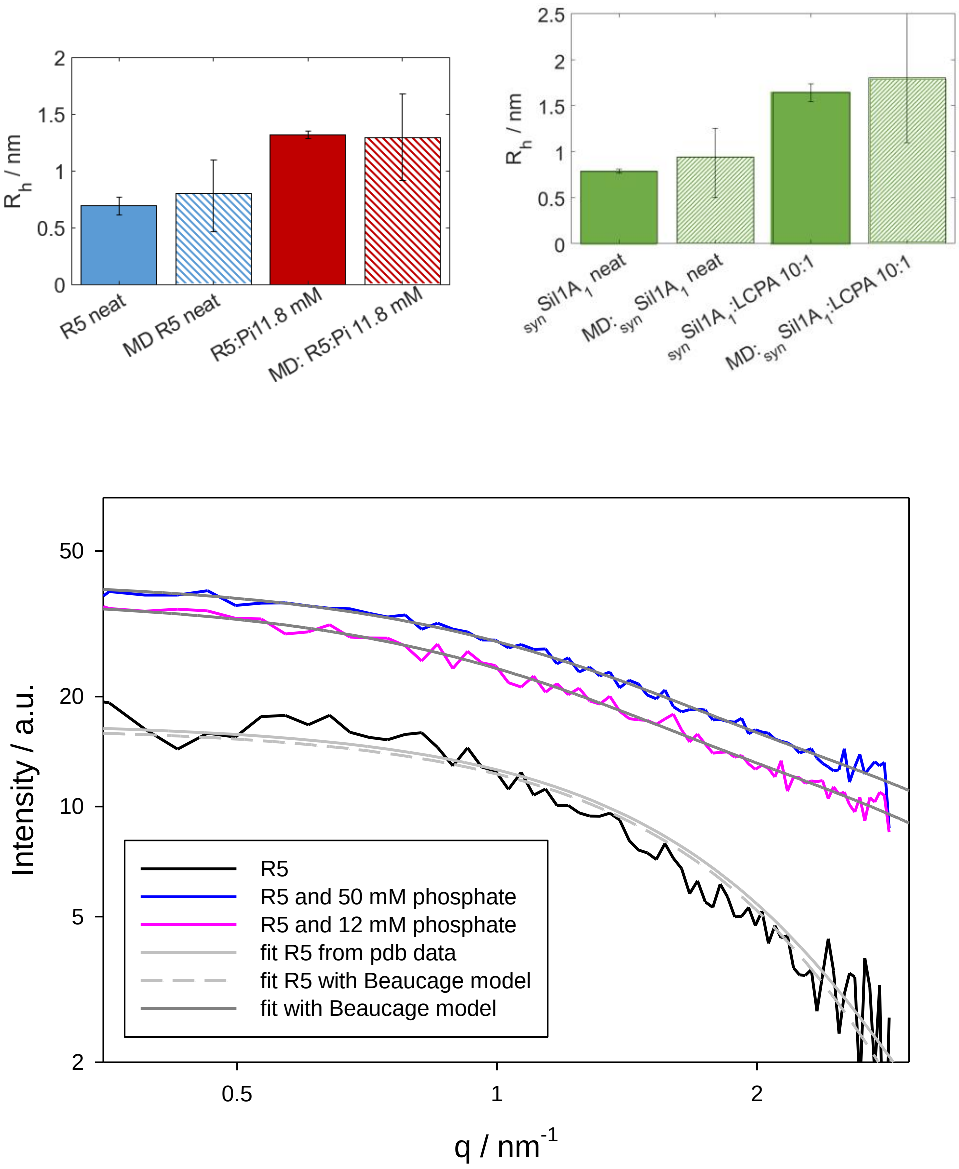

Fig. S9. Comparison for hydrodynamic radii determined either experimentally (solid colors) or in MD simulations (striped). The match is good within the precision of the experiments. The error bars correspond to the standard deviations found in the DOSY experiments or the R_h trajectories in the production MD runs. Bottom: SAXS intensities of R5 (black line), R5+11.8 mM phosphate (pink line), and R5+50mM phosphate (blue line). Fits from a pdb-model (see below) and Beaucage model for R5, light gray lines, and for the samples in phosphate with Beaucage model, dark gray lines.

Fig. S9 shows the scattering intensities of the three single components (R5, 11.8 mM phosphate, 50 mmol phosphate) after background subtraction. The fit curves (fit from pdb data, solid light gray line, from Beaucage model, dashed light gray line) for R5 (black line) are nearly

identical. A value for the radius of gyration of R_g = 0.9 nm was obtained, with an uncertainty of ±0.2 nm, estimated from varying fit parameters and the background.

In the case of both R5 and 12 and 50 mM phosphate, scattering intensities are considerably higher and differ from pure R5. As an increase of the scattering intensity is a consequence of a higher electron density difference, this clearly indicates that R5 and phosphate cooperatively form a different structure. A fit with the Beaucage model (dark gray lines) resulted in a slightly larger radius of R_g=1.3 nm for R5 in 50 mM phosphate and R_g = 1.4 nm for R5 in 11.8 mM phosphate, which are identical within the uncertainty of ±0.2 nm.

Due to the high background towards smaller q-values than q=0.35 nm^{-1}, corresponding to a size in real space of about 20 nm, no statement on the aggregation can be made. This range would be accessible to different techniques such as USAXS or light scattering techniques, where the latter was used in reference [4] additionally to SAXS and SANS measurements. In conclusion, the NMR and SAXS results on the size of R5 with and without phosphate coincide very well.

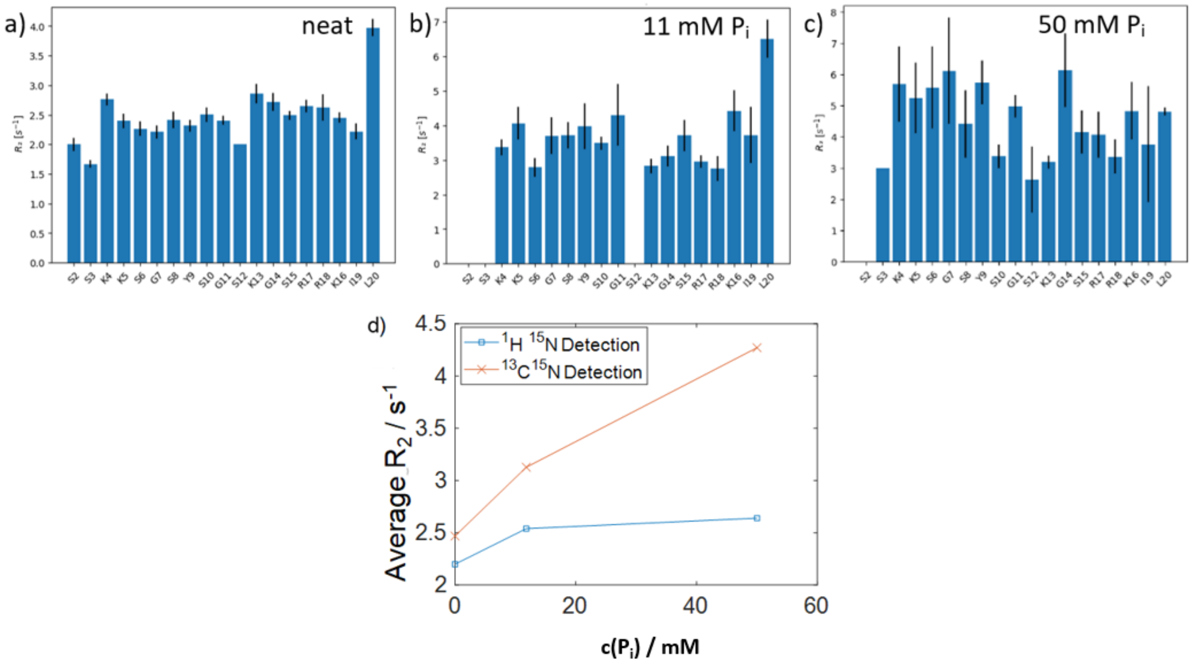

Figure S10. ^{15}N R_2 Relaxation rates measured by ^{13}C detected NMR for a) neat R5, b) R5 in 11.8 mM P_i and c) R5 in 50 mM P_i. d) Comparison of the average increase in R_2 at different P_i concentrations in ^{1}H and ^{13}C detection.

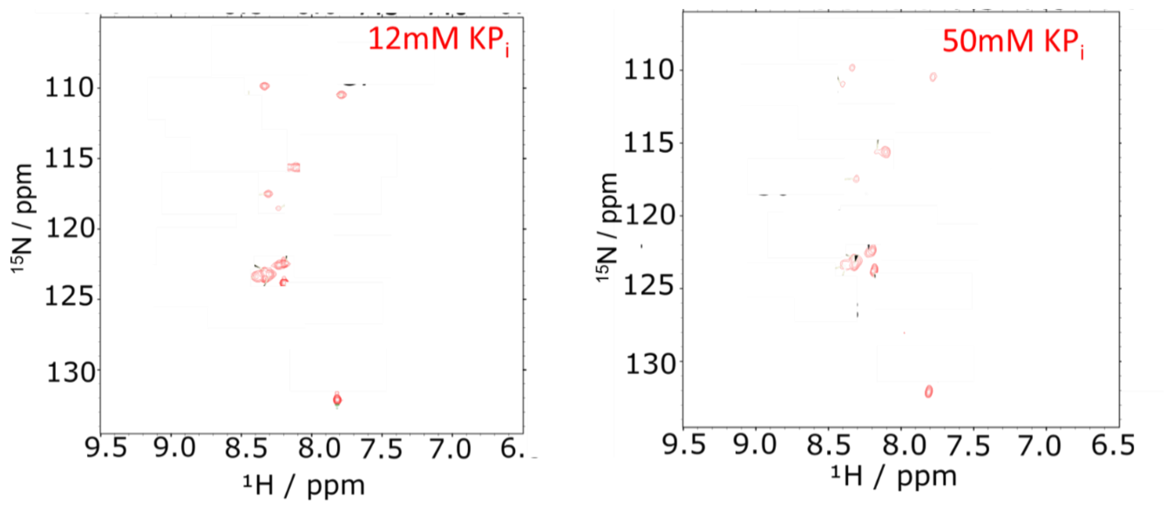

Fig. S11. ^{1}H-^{15}N HSQC for P_i concentrations of 11.8 mM (left) and 50 mM P_i (right). The signal intensities continue to drop due to the formation of assemblies, resulting in line broadening. It should be noted that for all observed resonances, no peak splitting was observed; only chemical shift perturbations were observed.

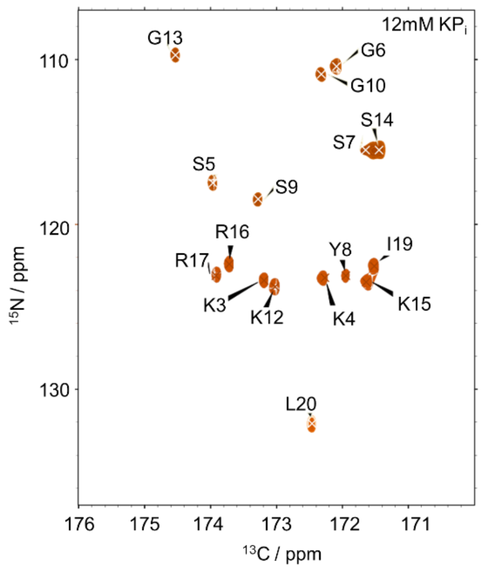

Fig. S12. ^{1}H^N-CON spectrum for R5 for the 11.8 mM P_i case.

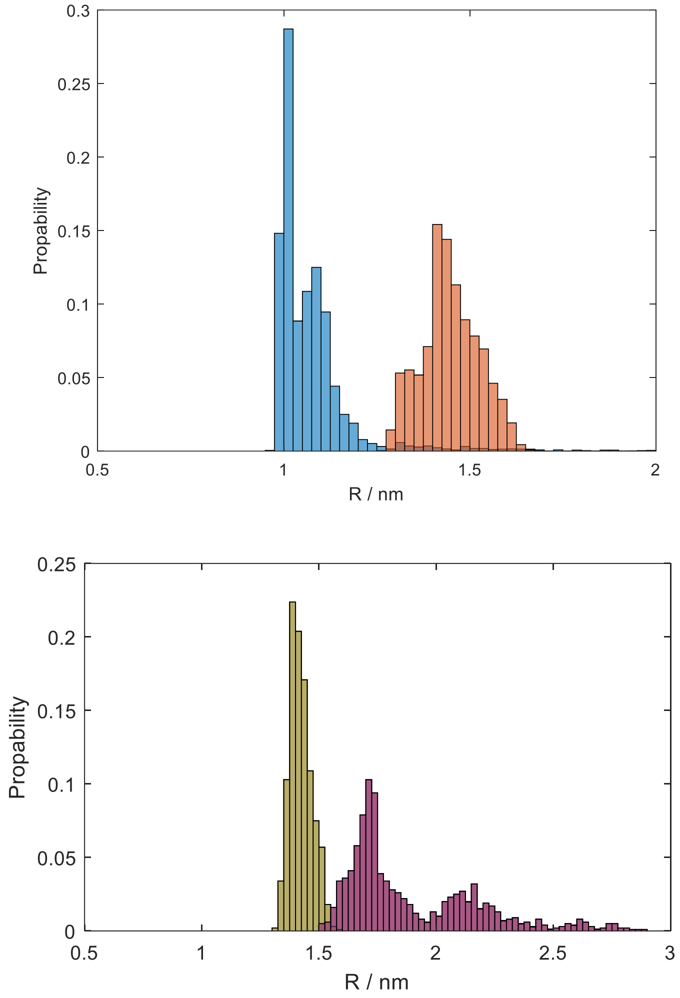

Figure S13. Comparison of radii sampled in MD simulations for R5 and *syn*Sil1-A_1 dimers (blue) and R5 trimers (red). Evidently, the dimer is significantly smaller. The difference is small (as expected by the referee) but significant. A Wilcoxon test yielded p-values of $7.3x10^{-3}$ and $1.8x10^{-4}$ for R5 and *syn*Sil1-A_1, respectively, ascertaining significantly different distance distributions. The sample size was 1000 for both peptides. All data were normalized to yield a total probability density of 1.

Supplementary MD Data

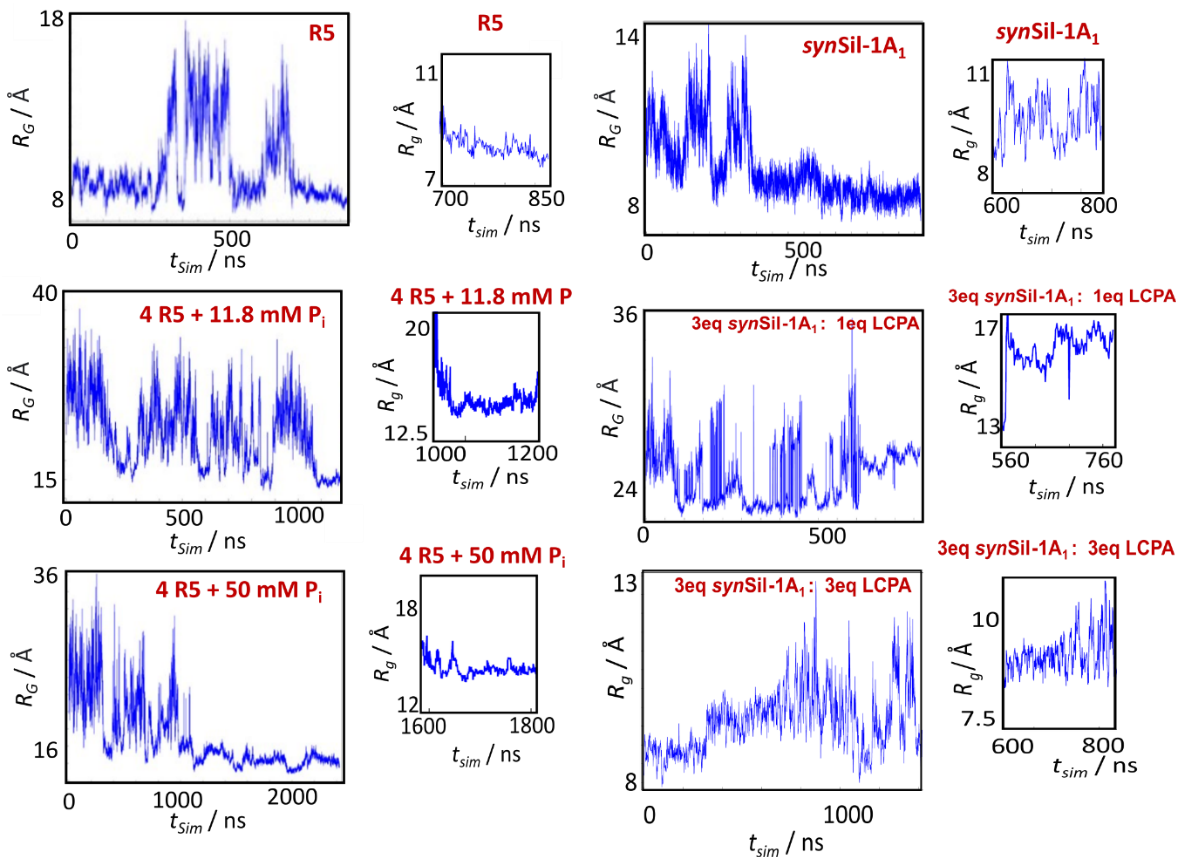

Fig. S14. Time traces of radii of gyration for different MD production runs. All replicas and further trajectories can be found in the supporting data sets.

For *syn*Sil-1A$_1$**,** the pulse sequence shown in Fig. S15 was created to detect ^{31}P transverse relaxation rates. As for R5, increasing relaxation rates (Fig. S16) upon exposure to growing LCPA counterion concentration indicate that more peptides self-assemble with rising ionic strength.

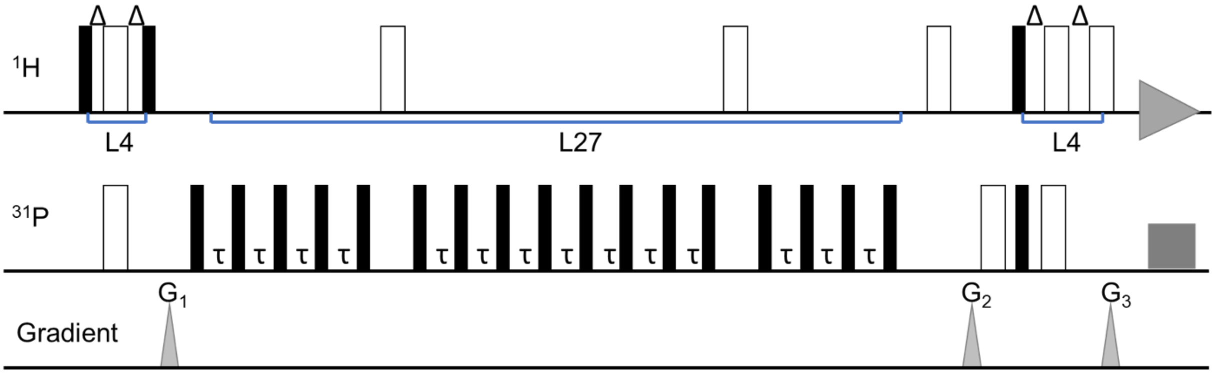

Fig. S15. Pulse-sequence scheme for ^{1}H-^{31}P HSQC with an additional CPMG pulse train for R_2 determination. Filled and wide rectangles represent 90° and 180° flip angle pulses, respectively. Delays of Δ = 100 µs and τ = 900 µs are applied as indicated. Loop L4 and Loop L27 were repeated 272 and 0 to 5 times, respectively. Gradient duration and levels were adjusted as follows, all with 1 ms duration: G_1= 5.35 G/cm, G_2= 42.80 G/cm, G_3= 17.33 G/cm.

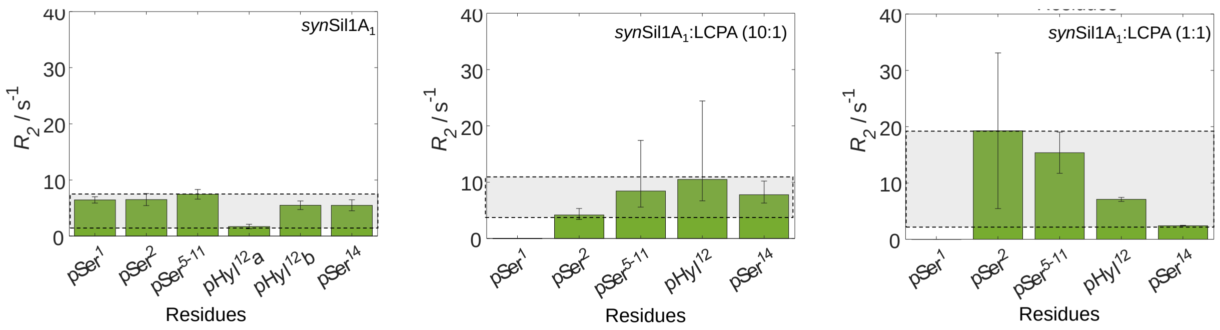

Fig. S16. ^{31}P-R_2 rate constants of *syn*Sil-1A$_1$ in vuffer (left) and in the presence of 0.1 and 1 equivalent of LCPA counterions (center and right). The grey shades indicate the range of observed values. The values were recorded using the pulse sequence shown in Fig. S15.

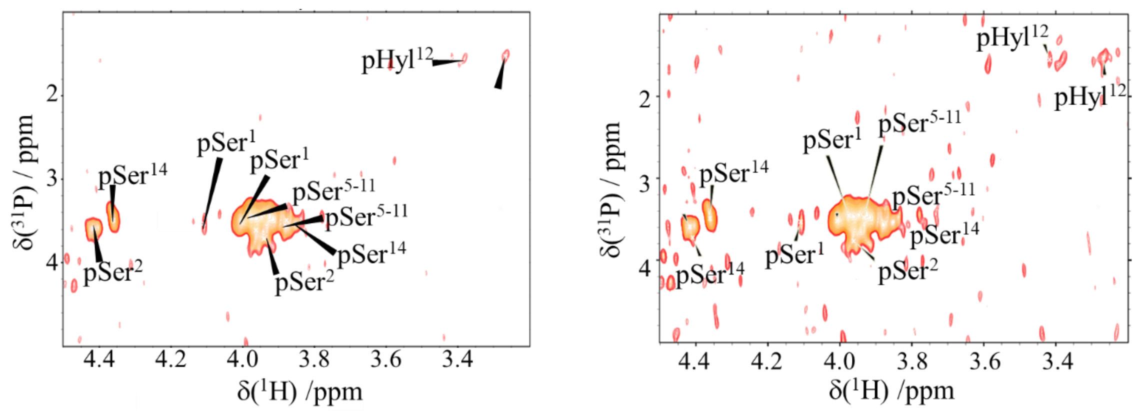

Fig. S17. ^{1}H-^{31}P HSQC for *syn*Sil-1A$_{1}$: LCPA 10:1 (left) and 1:1 (right). The signal intensities continue to drop due to the formation of PNS, which results in line broadening.

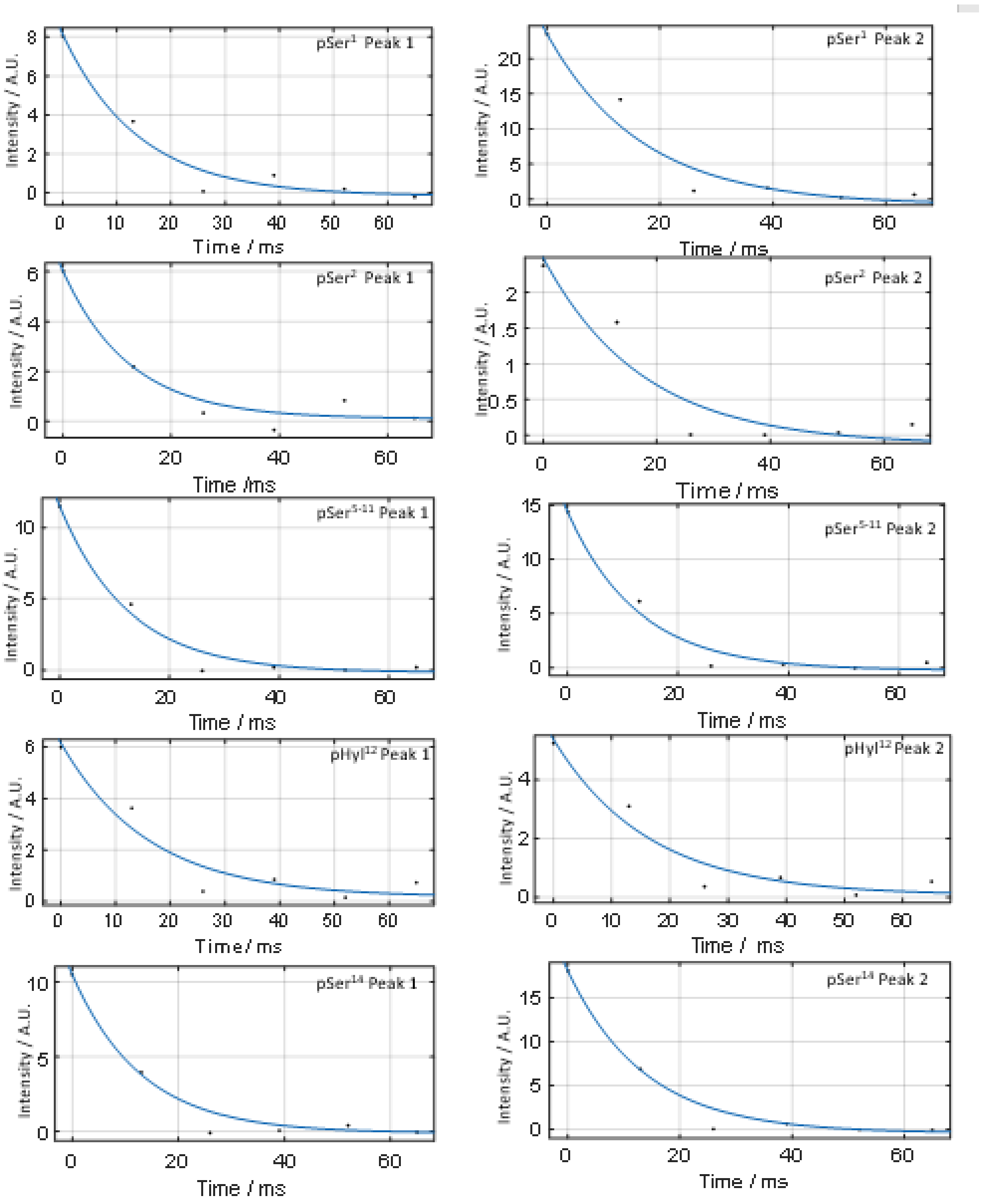

Fig. S18. ^{1}H-^{31}P T_2 relaxation fits of *syn*Sil-1A$_{1}$ in water.

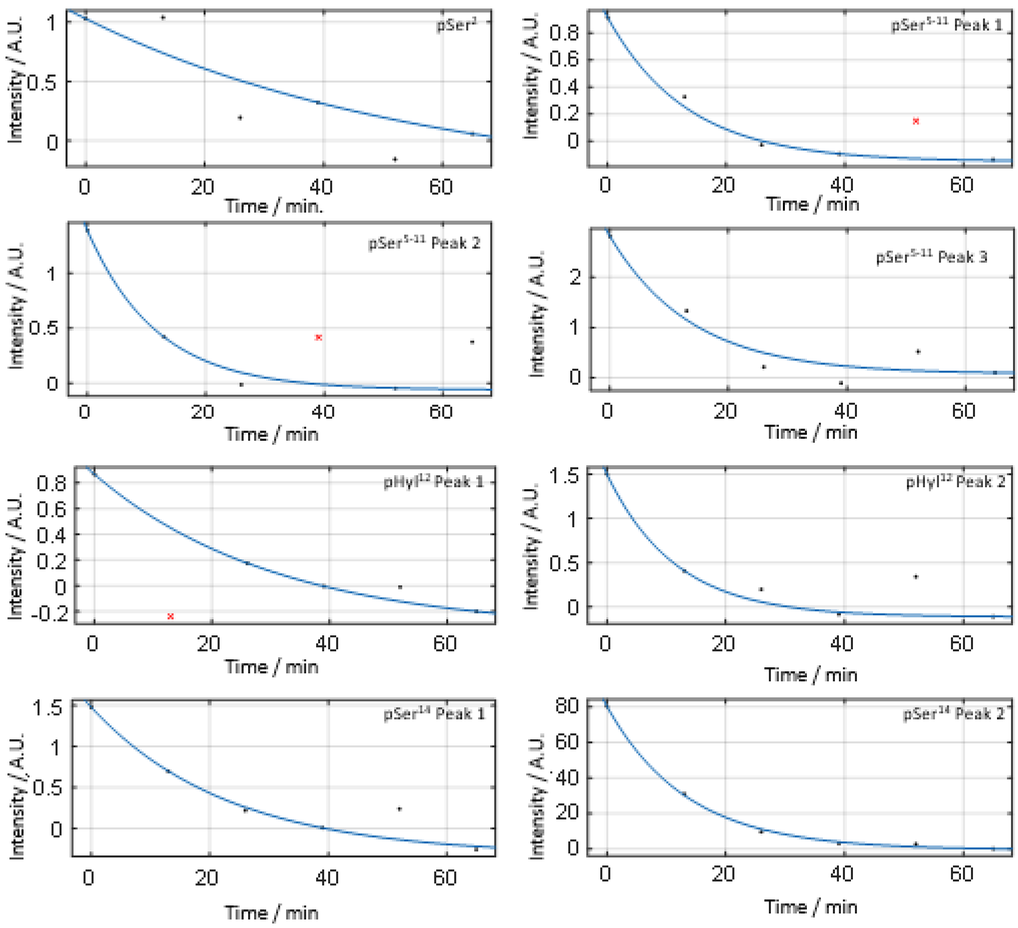

Fig. S19. ^{1}H-^{31}P T_2 relaxation fits of *syn*Sil-1A$_1$ and LCPA (10:1).

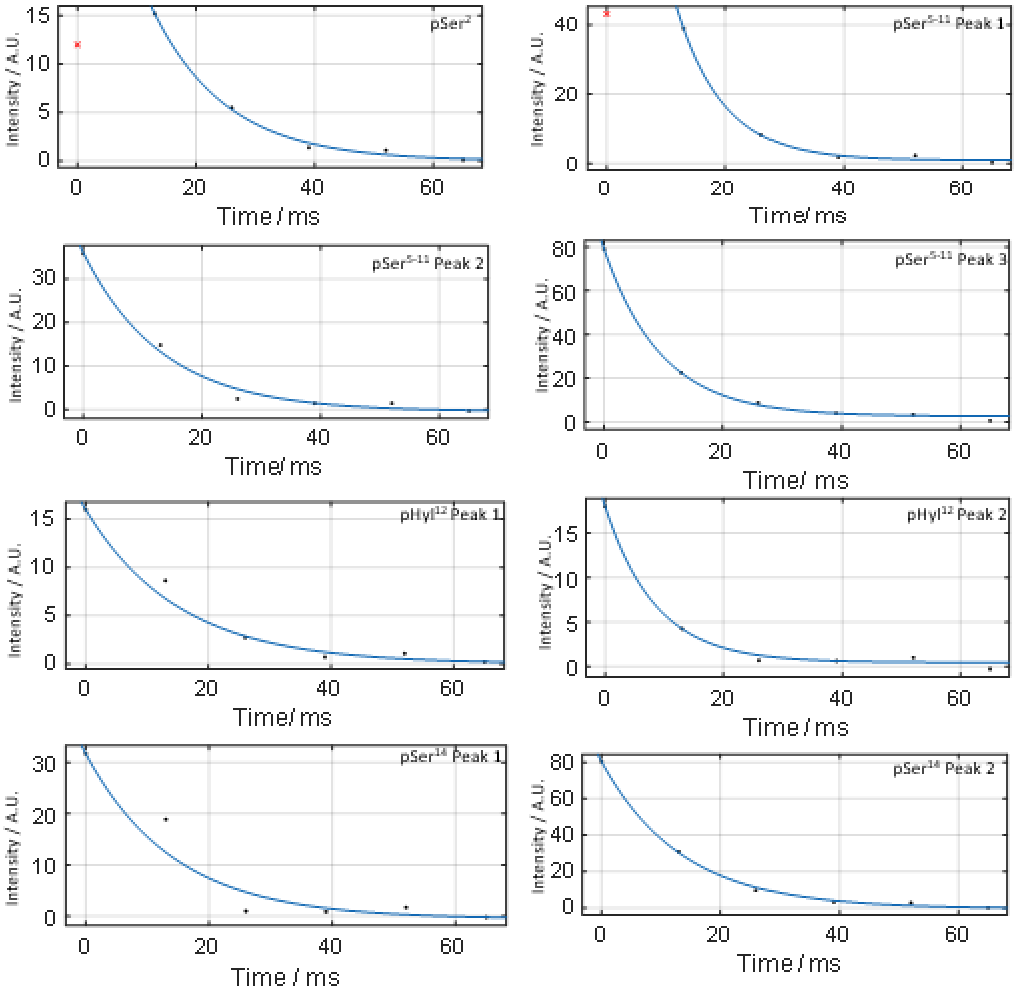

Fig. S20. ^{1}H-^{31}P T_2 relaxation fits of *syn*Sil-1A$_1$ and LCPA (1:1).

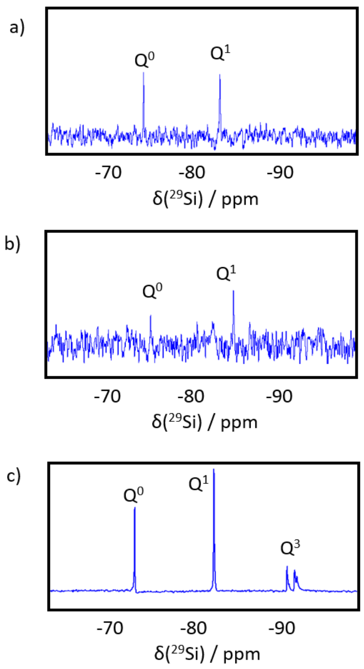

Figure S21. Solution-state ^{29}Si data recorded at different times of the silification process. a) Before the precipitation. Mono silicates and disilicates are visible. b) in the absence of any peptide, the equilibrium shifts towards disilicate within an hour. c) In the presence of R5, the equilibrium shifts to trifold substituted species in solution within a few minutes and then remains stable for hours.

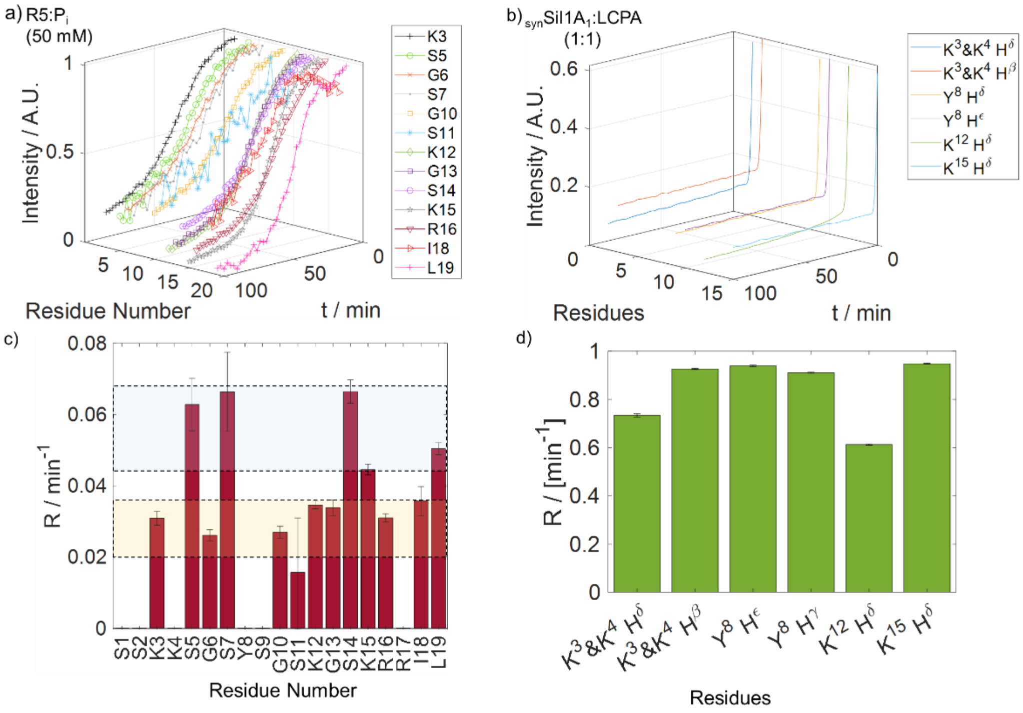

Fig. S22. a) Real-time precipitation assay with R5 in 50 mM P_i, tracing the intensity change in ^{1}H- ^{15}N SOFAST HMQC crosspeaks. b) Real time precipitation assay with *syn*Sil-1A$_1$: LCPA 1:1 molar ratio, tracing the intensity change in ^{1}H 1D spectroscopy. c) Per-residue exponential fit of rates for a). d) Sigmoidal fits for rates in different residue types in b). At high counterion ion concentrations of P_i and LCPA, respectively, the precipitation kinetics were too fast for real-time monitoring by NMR. Only the very slow equilibration of the system subsequent to particle formation is reflected by the data.

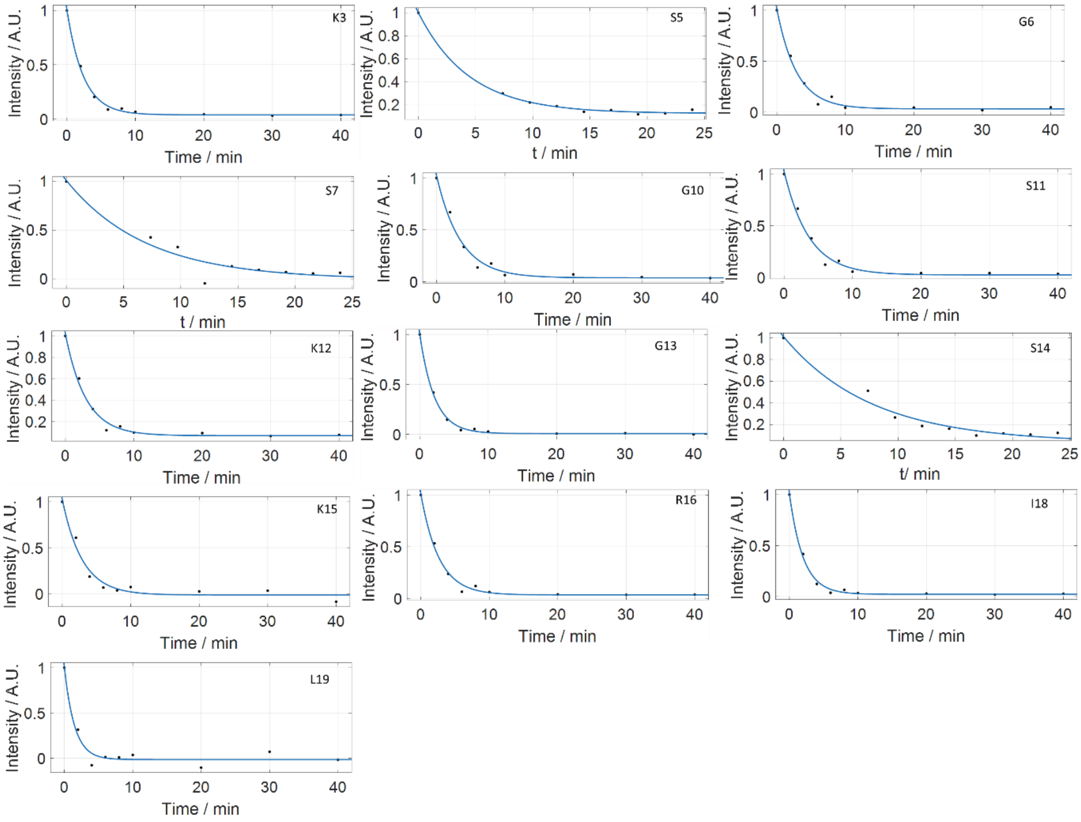

Fig. S23. Exponential fits of R5 (11.8 mM Pi) signal intensity decays upon silica formation.

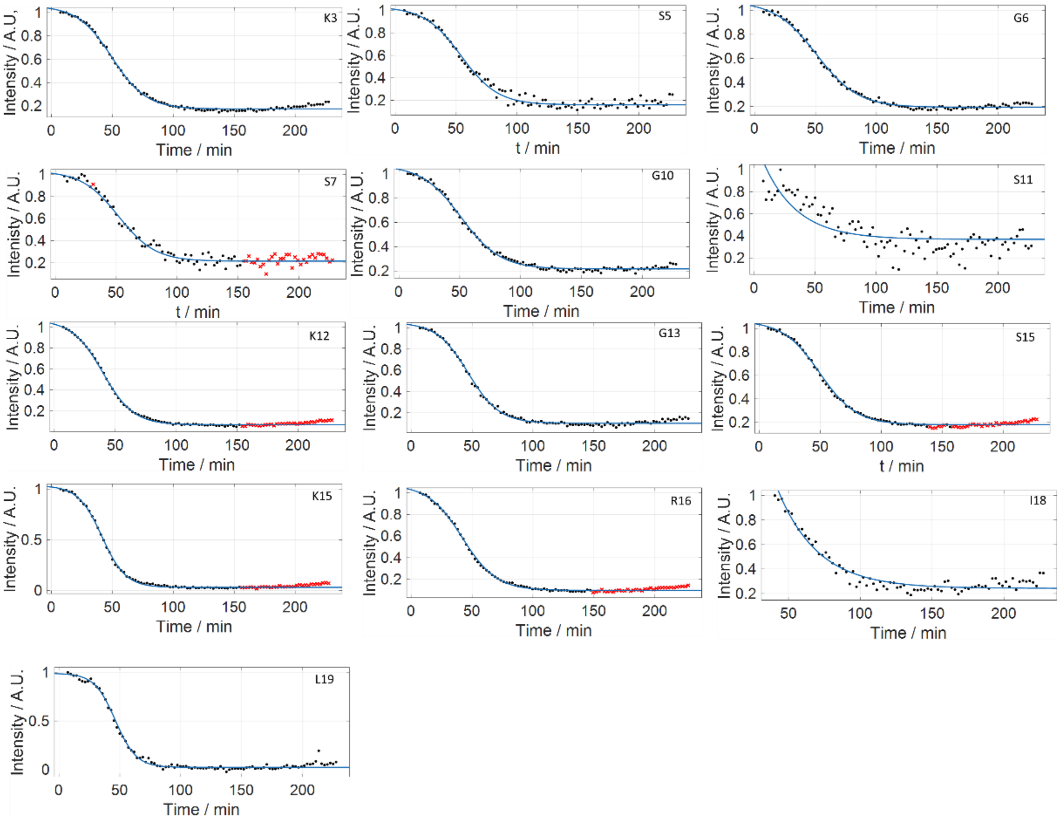

Fig. S24. Exponential fits of R5 (50 mM Pi) signal intensity decays upon silica formation.

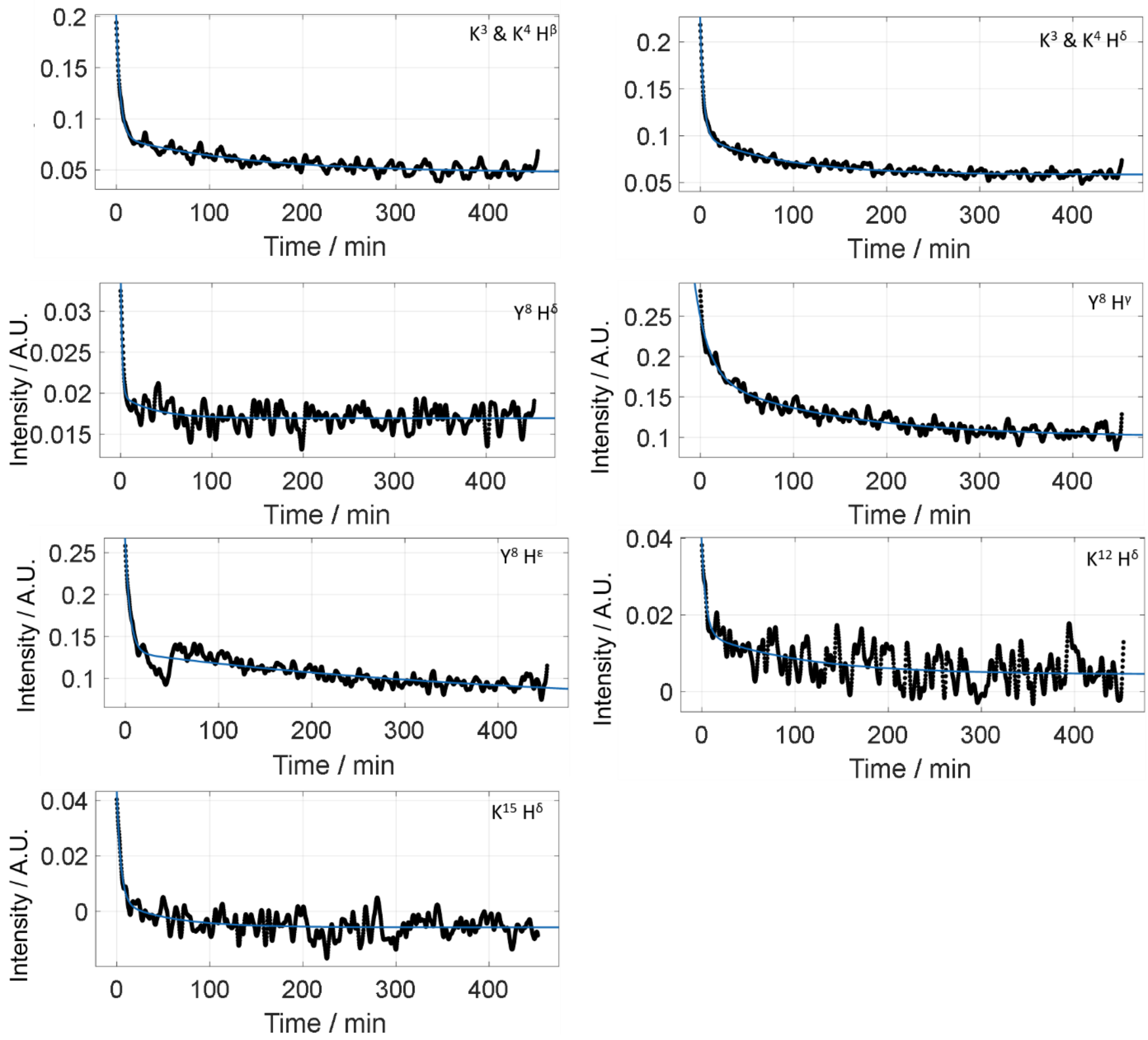

Fig. S25. Exponential fits of *syn*Sil-1A$_1$:LCPA 10:1 signal intensity decays upon silica formation.

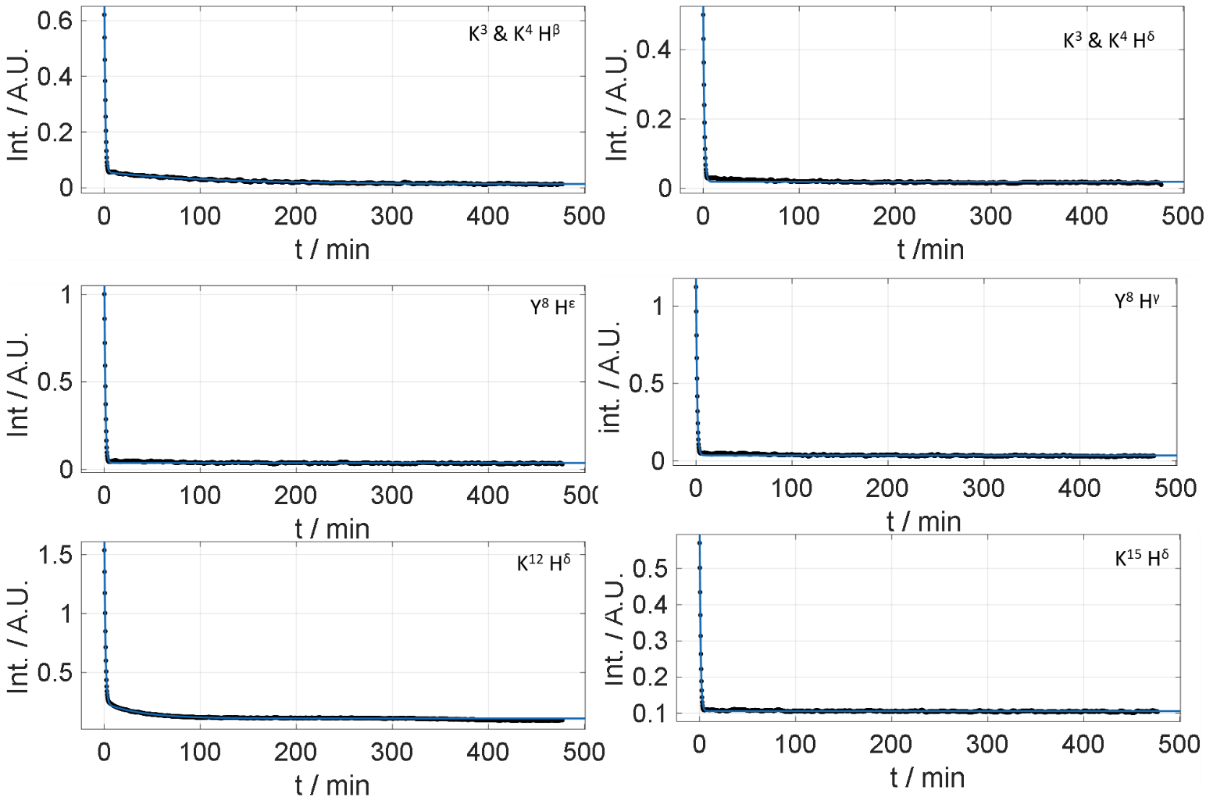

Fig. S26. Exponential fits of *syn*Sil-1A_1:LCPA 1:1 signal intensity decays upon silica formation.

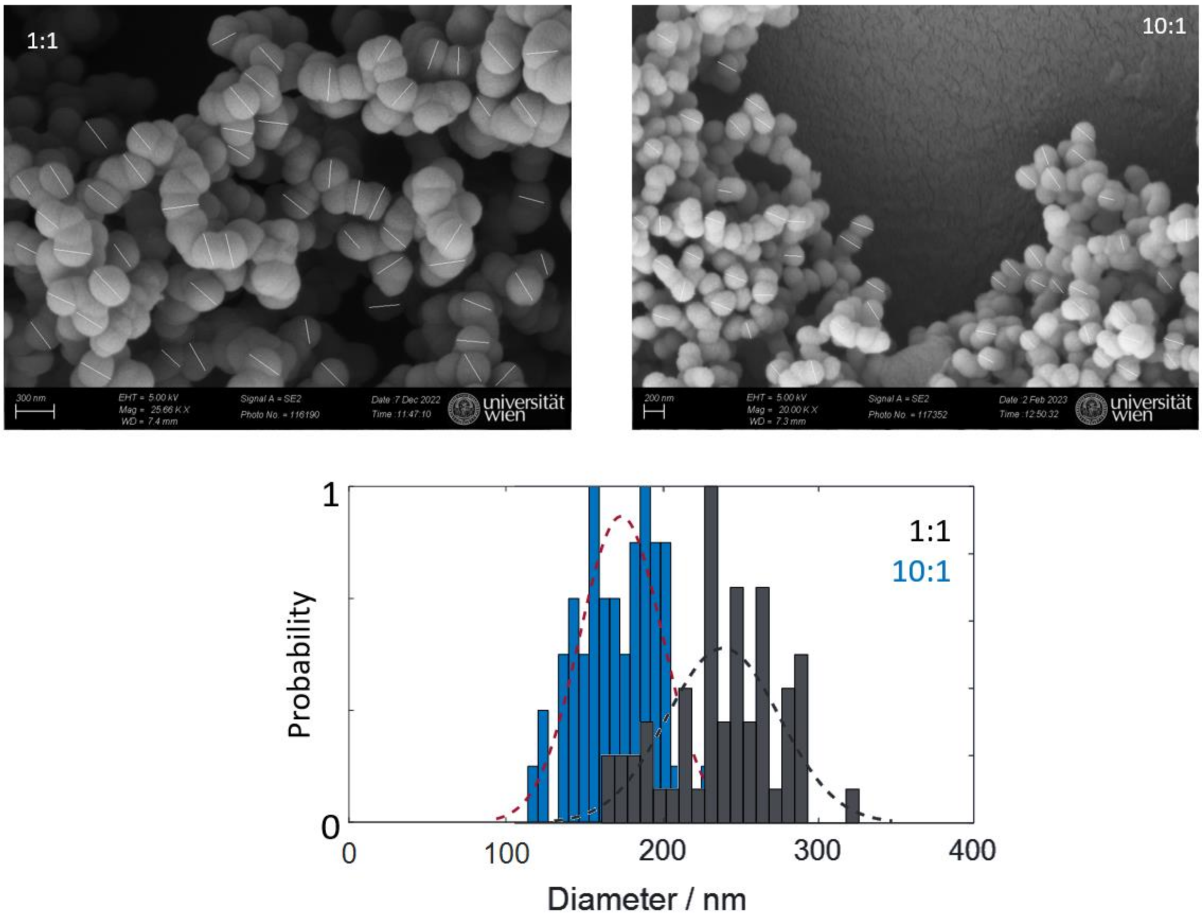

Figure S27. Top: *syn*Sil-1A$_1$-templated particles used to evaluate the particle diameters. The white stripes indicate where the diameters have been measured. Bottom: Size distributions and fits to normal distributions. Clearly, the particles grow with increasing LCPA content.

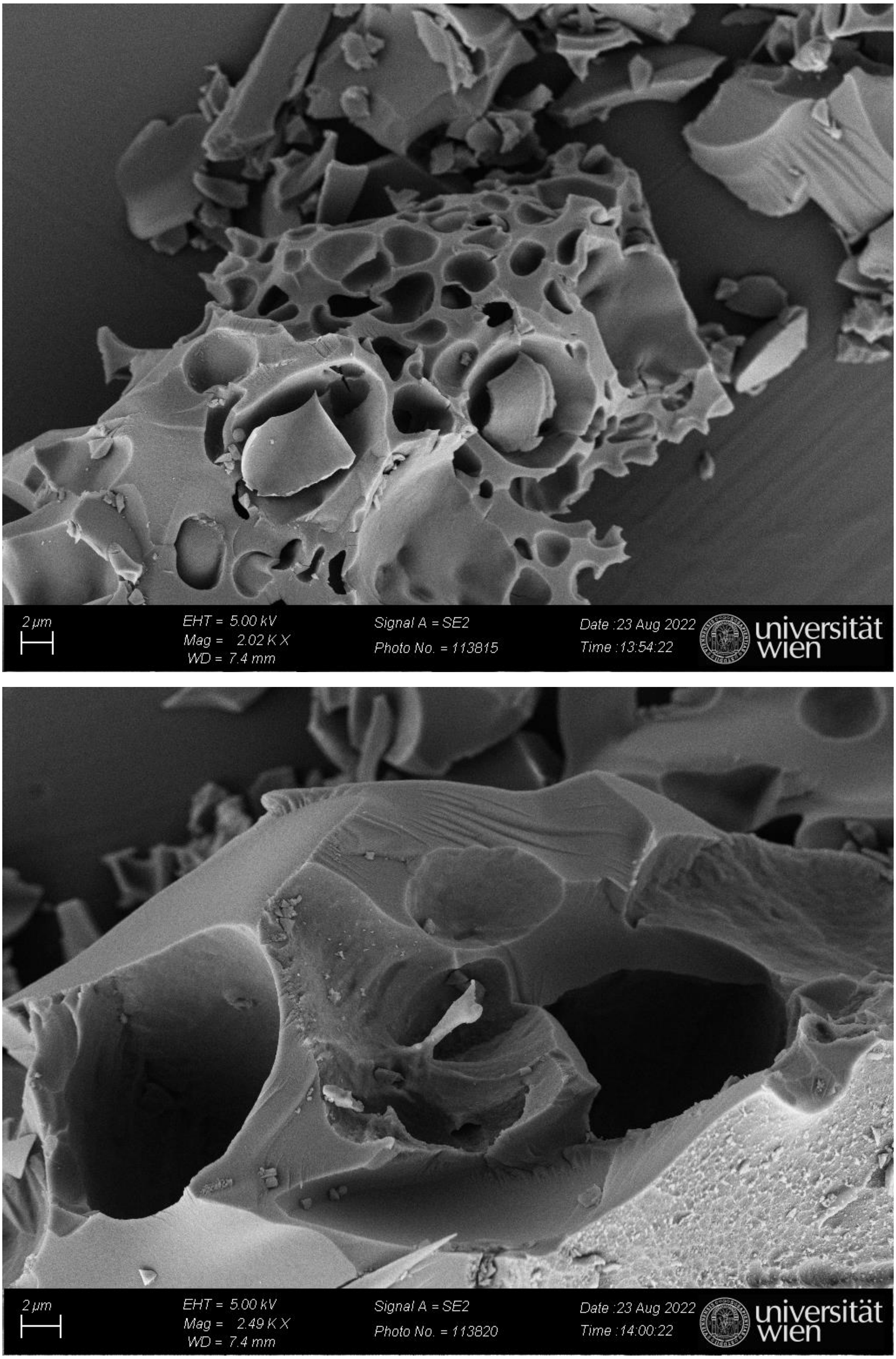

Fig. S28. SEM of silica precipitated in the absence of any peptide over a duration of 2 days. No defined structure is observable.

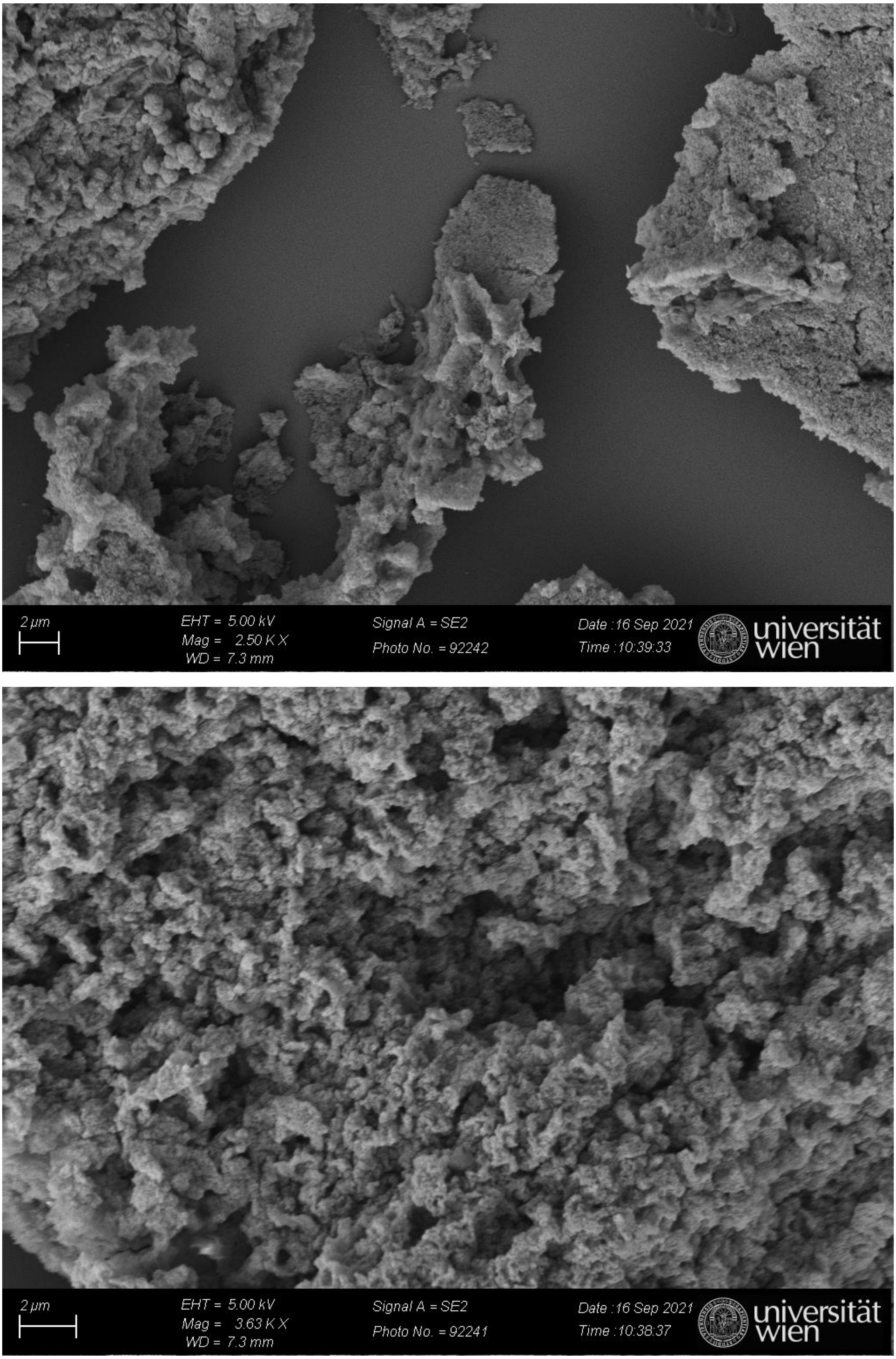

Fig. S29. Additional SEM images of silica precipitate templated by R5 (11.8 mM P_i).

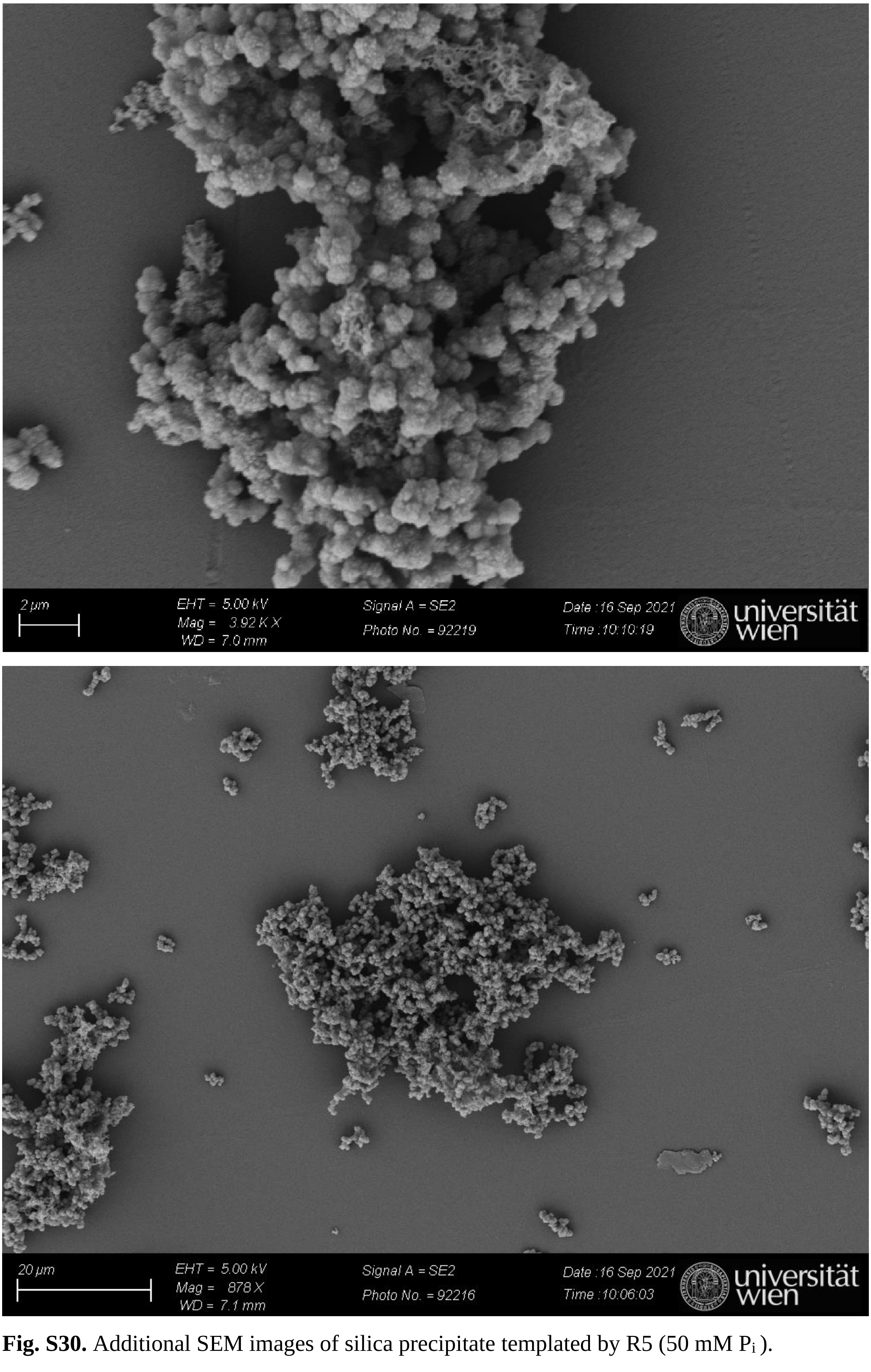

Fig. S30. Additional SEM images of silica precipitate templated by R5 (50 mM P_i).

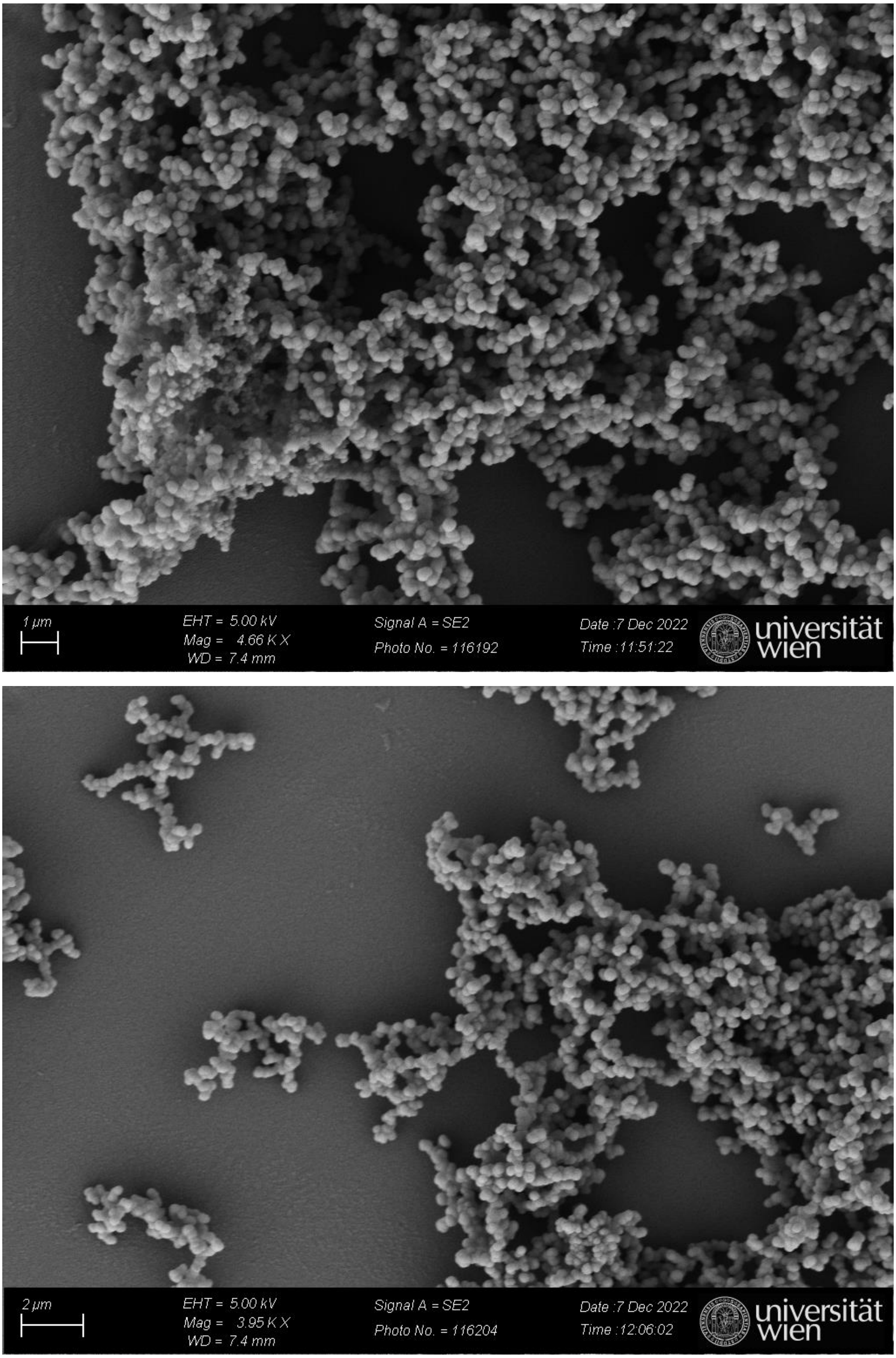

Fig. S31. Additional SEM images of silica precipitate templated by *syn*Sil-1A_1 and LCPA at a molar ratio of 1:1.

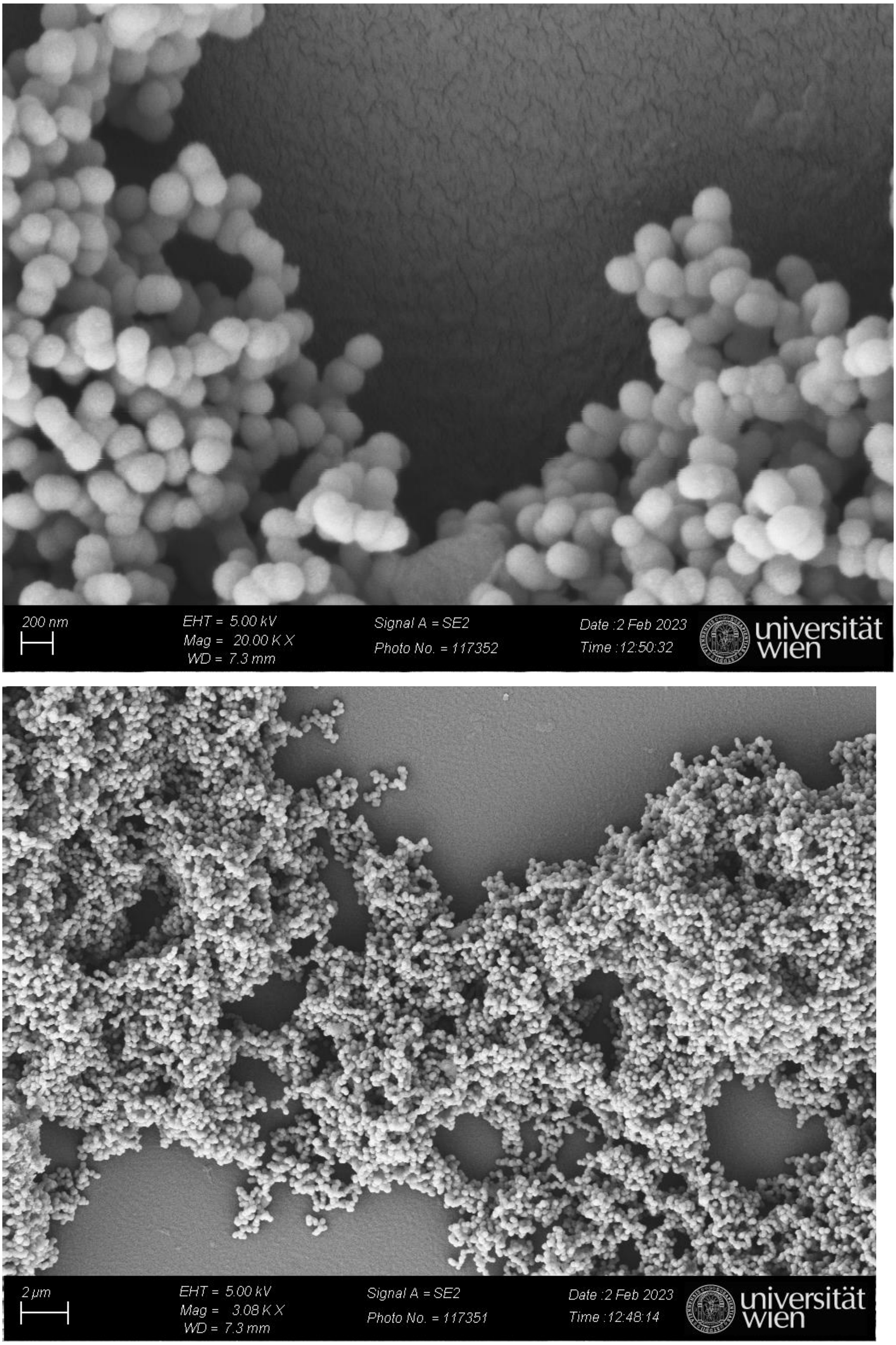

Fig. S32. Additional SEM images of silica precipitate by synSil-1A_1 and LCPA at a molar ratio of 10:1.

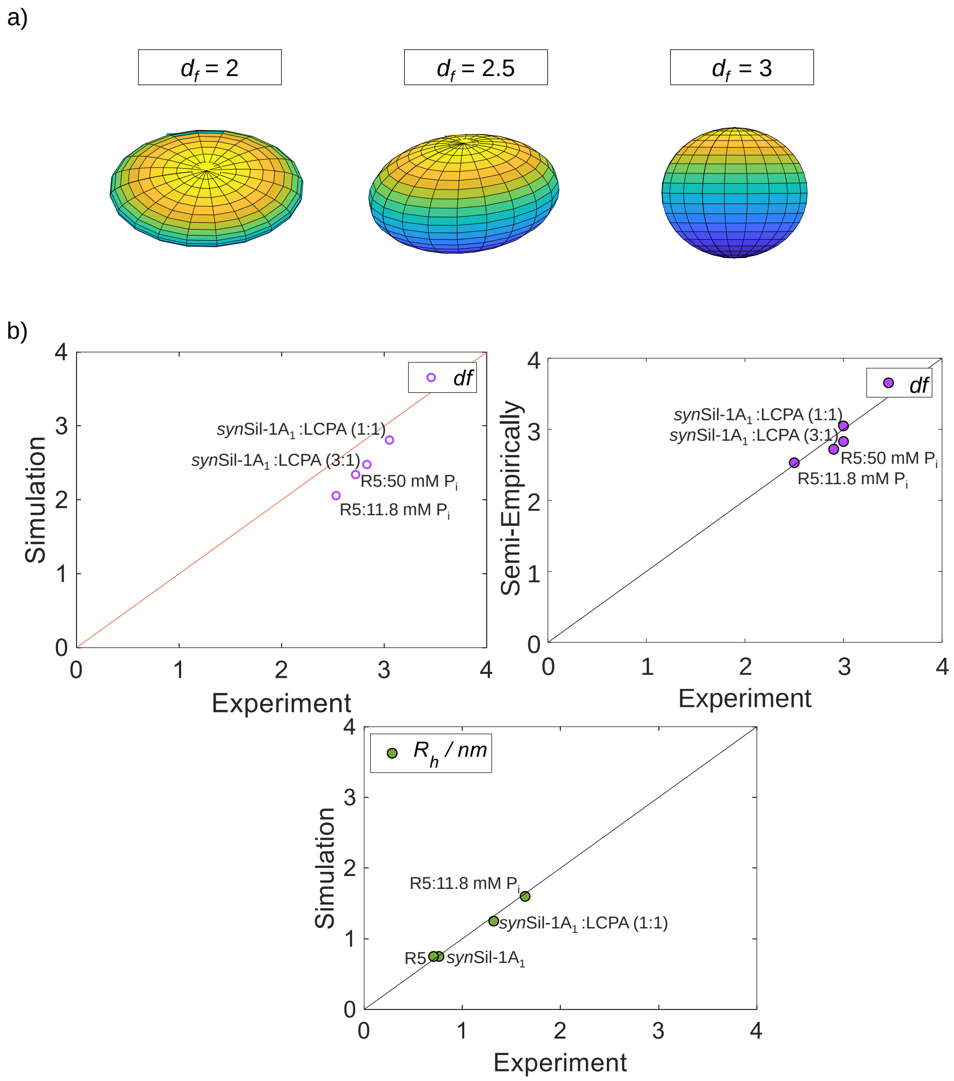

Fig. S33. a) Scheme of how different fractal dimension values (d_f) represent different structures. Note that this representation is for visualization purposes only and is not an actual representation of the simulated systems. b) Top: Correlation plot for experimental and MD-simulated as well as semi-empirically (rescaled R_g used from experimental R_h) derived fractal dimension (d_f, purple dots; d_f is unitless) of the four R5 and synSil-1A$_1$ self-assemblies. Bottom: Correlation of hydrodynamic radii, rescaled from calculated R_g, of the peptide trimers from experiments and simulations.

For R5 self-assemblies in solution, a fractal dimension of d_f = 2.5 at low P_i counterion concentrations has been determined in earlier studies[4,] indicating a somewhat planar morphology (the limiting case of d_f = 2 would correspond to a 2D object, d_f = 3 to spherical objects)[5]. Well-aligning with these observations, the silica coating of our R5 self-assemblies under these conditions led to silica plates with an identical fractal dimension of 2 <d_f < 3 upon silica co-precipitation (Fig. 8a-b). In contrast, at high counterion concentrations, a self-assemblies d_f of ~3 was observed earlier.[4] Accordingly, these assemblies led to spherical silica

particles in our SEM experiments, again with a d_f similar to that of the self-assemblies (Fig 8a-d). Hence, the fractal dimension of the self-assemblies seems to predetermine the fractal dimension, *i.e.*, the structure of the solid silica particles. For *syn*Sil-1A$_1$, we found d_f = 3 for both probed counterion concentrations, *i.e.*, spherical particles appeared in both cases. However, different particle sizes were found for the two different conditions in agreement with earlier reports[6]. Hence, while the fractality of the self-assemblies predetermines the shape of the silica particles for R5, the size of the self-assemblies governs the size of the final particles for *syn*Sil-1A$_1$.

To validate this finding, the experimental fractal dimensions were compared with those obtained by our MD simulations. To determine d_f, we employed the method described by Polyansky *et al.* in reference [7.8]. We used Eqs. 1-13 reported therein[7,8] for our calculations. All values used for the calculations are shown in Table S2. For R5, we found a fractal dimension df = 2.53 at low concentrations, which increased to 2.72 upon increasing the P_i concentration. Hence, the particle dimensionality observed in the electron micrographs, as well in the reported literature for the peptide self-assemblies before coating, correlated well with the simulations. Similar good matches were found for *syn*Sil-1A$_{1.}$ While d_f = 2.83 was found at low LCPA concentrations, 3.05 was computed for high LCPA concentrations (calculated by rescaled Rg from experimentally derived R_h). Fig. S33 shows the correlation between simulated and experimentally determined hydrodynamic radii of the tripeptide subunits and the fractal dimension of all studied self-assemblies. Overall, the match corroborates the simulated structures.

The slight underestimation of peptide compactness in simulations (Fig. S33) might be related to the difference in compactness estimation suggested in Polyansky *et al.*[7,8] Herein, the assemblies are constituted by timer-building blocks with a different R_g as compared to individual peptides.

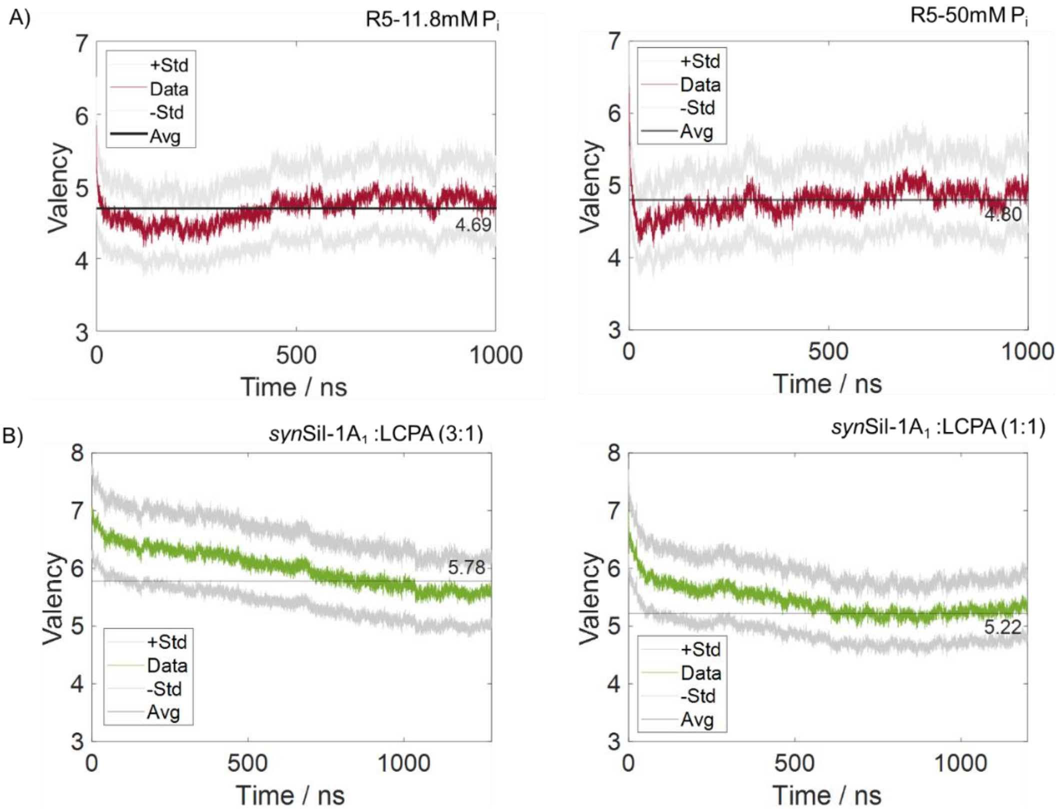

Fig. S34. Time traces of interaction valency in crowded-state simulations for different systems. A) R5 in 11.8 mM P_i (left) and R5 in 50 mM P_i (right). B) *syn*Sil-1A_1 :LCPA (3:1) (left) and *syn*Sil-1A_1 :LCPA (1:1) (right).

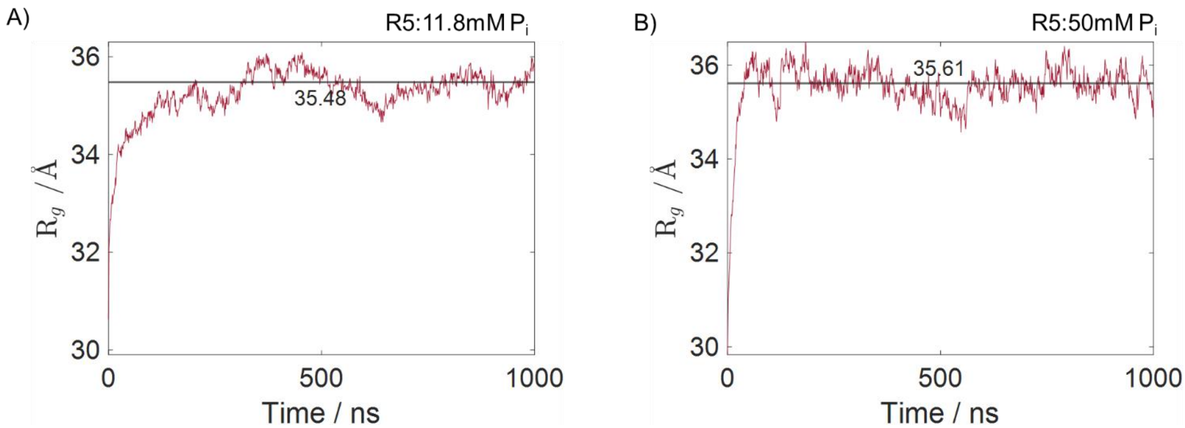

Fig. S35. Time traces of the overall radius of gyration for the simulated assemblies of R5 with (A) 11.8 mM P_i and (B) 50 mM P_i (B). Average over 25 R5 units.

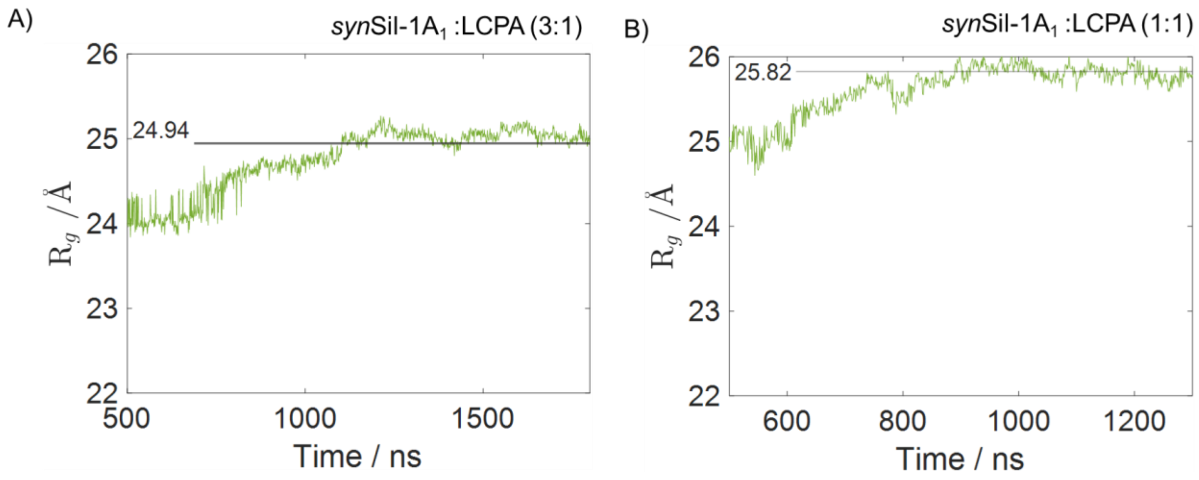

Fig. S36. Time traces of the overall radius of gyration for the simulated assemblies of 25 *syn*Sil-$1A_1$ units with different concentrations of LCPA for (A) synSil-$1A_1$:LCPA (3:1) and (B) synSil-$1A_1$:LCPA (1:1), Average over all 25 units.

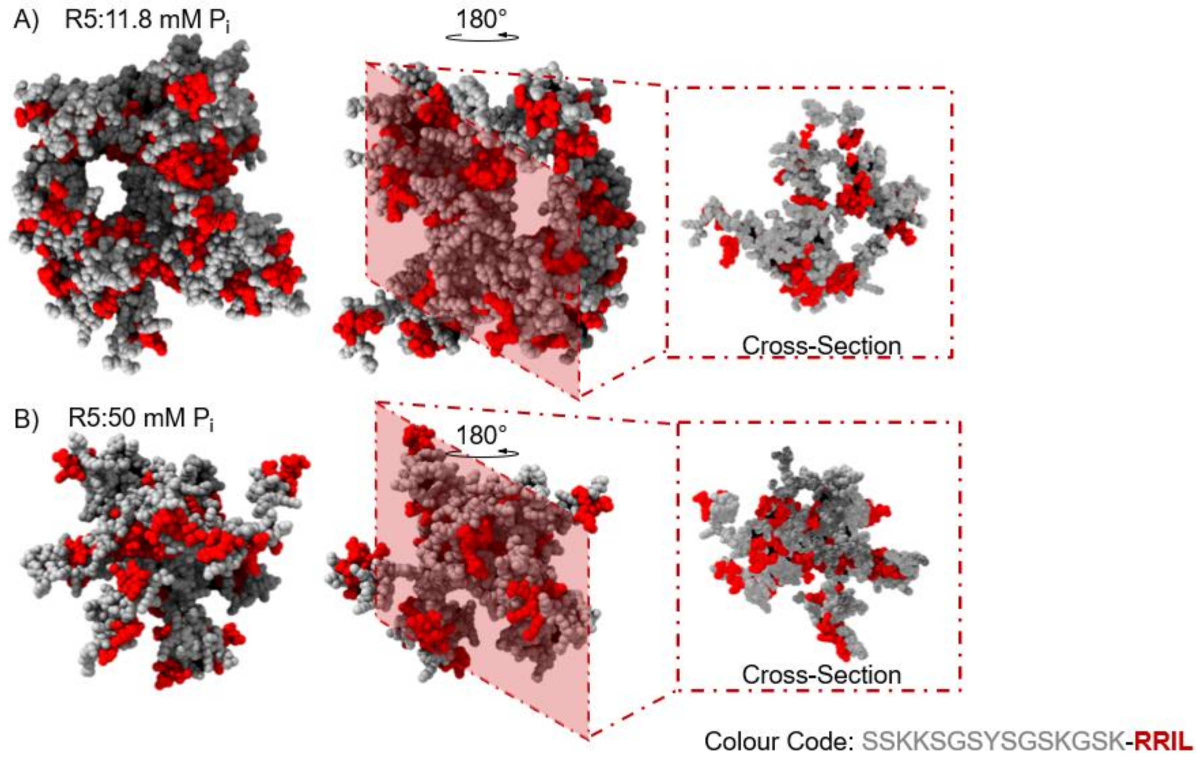

Fig. S37. Many of the RRIL motifs are located at the surface, which is also in line with our earlier observation showing that the RRIL motif is involved in silica scavanging[9]. However, some of these motifs are buried within the structure, likely providing stability to the self-assemblies.

Further Tabulated Data

Table S1. NMR resonance ssignments for ^{13}C and ^{15}N labeled R5. The assignment was achieved by combining HNCACB, HNN, and HSQC spectra.

	^{13}C-Cα [ppm]	^{13}C-Cβ [ppm]	^{15}N [ppm]	^{1}H [ppm]
S2	58.305	63.76	117.024	8.295
K3	57.308	38.852	119.849	7.166
K4	56.737	33.039	119.889	7.203
S5	59.538	64.178	117.818	8.396
G6	45.503	-	110.941	8.459
S7	58.38	63.998	115.608	8.189
Y8	61.018	38.192	122.759	8.327
S9	58.268	63.895	118.777	8.299
G10	45.498	-	110.433	7.822
S11	58.504	64.153	115.57	8.227
K12	57.084	33.306	123.641	8.419
G13	45.475	-	109.831	8.397
S14	58.299	63.767	115.621	8.164
K15	56.124	31.011	123.335	8.379
R16	56.296	31.173	122.629	8.275
R17	57.1	32.612	123.576	8.469
I18	56.47	32.945	123.918	8.267
L19	56.555	42.874	130.808	8.056

Table S2. Overview of parameters extracted from MD simulations to compute the peptide structures and trimer structures:

Simulated System	Number of Peptides	Number of Water Atoms	Na^+	Cl^-	Box size [Å]	Simulation length [ns]
R5 neat	1	3265	6	12	46.50	700
R5: Pi (11.8 mM)	1	8508	20	27	63.70	1097.20
R5: Pi (50 mM)	1	894	-	17	31.50	1932.40
4R5: Pi (11.8 mM)	4	8324	6	34	63.86	774.60
4R5: Pi (50 mM)	4	8149	6	34	63.60	2430.60
*syn*Sil-1A_1	1	2484	1	-	42.55	784.30
*syn*Sil-1A_1:LCPA (3:1)	1	1770	12	4	39.42	715.50
*syn*Sil-1A_1:LCPA (1:1)	1	3762	4	1	48.7	952
*syn*Sil-1A_1:LCPA (3:3)	3	6886	23	24	59.93	763

Table S3. Overview for parameters extracted from MD simulations to compute the objects' fractal dimensions see Methods, Valency was determined by monomer:

Simulated System	Number of Peptides	Number of Water Atoms	Na^+	Cl^-	Box size [Å]	Simulation length [ns]	Density [g/cm^3]	Valency
R5: Pi (11.8 mM)	25	8879	2	432	72.6	1090.50	0.6100	4.81 ± 0.18
R5: Pi (50 mM)	25	7266	-	425	71.5	1092.80	0.6194	4.92 ± 0.18
*syn*Sil-1A$_1$: LCPA (3:1)	25	2897	5	5	52.0	1008.20	0.8899	5.57 ± 0.33
*syn*Sil-1A$_1$: LCPA (1:1)	25	2326	5	5	57.0	1305.60	0.9754	5.22 ± 0.28

Bruker TopSpin 4 Pulse program for ^{1}H- ^{31} P CPMG-HSQC

```
;na_hsqcetf3gpxy.go adapting for 2-channels from Bruker seq. na_hsqcetf3gpxy
;avance-version (12/01/11)
;HSQC
;2D H-1/P-31 correlation
;   using XY16-CPMG for transfer
;phase sensitive using Echo/Antiecho-TPPI
;using f3 - channel
;(use parameterset NA_HSQCETF3GPXY)
;
;B. Luy & J.P. Marino, J. Am. Chem. Soc. 123, 11306-11307 (2001)
;(F.A.A. Mulder, C.A.E.M. Spronk, M. Slijper, R. Kaptein &
;   R. Boelens, J. Biomol. NMR 8, 223-228 (1996))
;(Davis et al., J. Magn. Reson. 98, 207 - (1992))
;
;$CLASS=HighRes
;$DIM=2D
;$TYPE=
;$SUBTYPE=
;$COMMENT=
prosol relations=<triple_na>
#include <Avance.incl>
#include <Grad.incl>
#include <Delay.incl>
"p2=p1*2"
"p22=p21*2"
"d11=30m"
"d26=1s/(cnst4*4)"
"d0=3u"
"in0=inf1/2"
"DELTA1=p16+d16+4u"
"COUNTER=d26*2/((d21*2+larger(p2,p22))*16)"
"l4=COUNTER*16"
"d31=(d21*2+larger(p2,p22))*l4"
```

```
1 ze

  d11 pl16:f2
2 d1 do:f2
3 50u pl3:f2 UNBLKGRAD
 20u rpp6
  20u rpp16
  (p1 ph1)
4 d21
  (center (p2 ph6) (p22 ph16):f2 )
  d21 ipp6 ipp16
  lo to 4 times l4
  (p1 ph2)
  4u
  p16:gp1
  d16
  (p21 ph3):f2
  d0
  (p2 ph5)
  d0
  p16:gp2*EA
  d16
  (p22 ph4):f2
  DELTA
  (ralign (p1 ph1) (p21 ph4):f2 )
5 d21
  (center (p2 ph6) (p22 ph16):f2 )
  d21 ipp6 ipp16
  lo to 5 times l4
  DELTA1
  (p2 ph1)
  p16:gp3

  d16 pl16:f2
  4u BLKGRAD
```

```
  go=2 ph31 cpd3:f2
  d1 do:f2 mc #0 to 2
    F1EA(calgrad(EA), caldel(d0, +in0) & calph(ph3, +180) & calph(ph31, +180))
  d31
exit

ph1=0
ph2=1
ph3=0 2
ph4=0 0 0 0 2 2 2 2
ph5=0 0 2 2
ph6=0 1 0 1 1 0 1 0 2 3 2 3 3 2 3 2
ph16=0 1 0 1 1 0 1 0 2 3 2 3 3 2 3 2
ph31=0 2 0 2 2 0 2 0

;pl1 : f1 channel - power level for pulse (default)
;pl2 : f2 channel - power level for pulse (default)
;pl3 : f3 channel - power level for pulse (default)
;pl12: f2 channel - power level for CPD/BB decoupling
;pl16: f3 channel - power level for CPD/BB decoupling
;sp13: f2 channel - shaped pulse 180 degree (adiabatic)
;spnam13: Crp60,0.5,20.1
;p1 : f1 channel -  90 degree high power pulse
;p2 : f1 channel - 180 degree high power pulse
;p8 : f2 channel - 180 degree shaped pulse for inversion (adiabatic)
;     = 500usec for Crp60,0.5,20.1
;p16: homospoil/gradient pulse                    [1 msec]
;p21: f3 channel -  90 degree high power pulse
;p22: f3 channel - 180 degree high power pulse
;d0 : incremented delay (2D)                      [3 usec]
;d1 : relaxation delay; 1-5 * T1
;d11: delay for disk I/O                          [30 msec]
;d16: delay for homospoil/gradient recovery
;d21: delay in XY16-CPMG                          [100 usec]
;d26: 1/(4J(PH))
```

```
;d31: 1/(2J(PH)) as executed
;cnst4: = J(PH)                              [20 Hz]
;o2p: 77 ppm
;o3p: 0 ppm
;l4: loop for XY16-CPMG
;inf1: 1/SW(P) = 2 * DW(P)
;in0: 1/(2 * SW(P)) = DW(P)
;nd0: 2
;ns: 4 * n
;ds: >= 32
;td1: number of experiments
;FnMODE: echo-antiecho
;cpd2: decoupling according to sequence defined by cpdprg2
;cpd3: decoupling according to sequence defined by cpdprg3
;pcpd2: f2 channel - 90 degree pulse for decoupling sequence
;pcpd3: f3 channel - 90 degree pulse for decoupling sequence
;use gradient ratio:     gp 1 : gp 2 : gp 3
;                          50 :   80 : 32.4    for P-31
;for z-only gradients:
;gpz1: 10%
;gpz2: 80%
;gpz3: 32.4% for P-31
;use gradient files:
;gpnam1: SMSQ10.100
;gpnam2: SMSQ10.100
;gpnam3: SMSQ10.100
;$Id: na_hsqcetf3gpxy,v 1.6 2015/07/20 11:20:57 ber Exp $
```

Pulse program code for ^{1}H- ^{31} P CPMG-HSQC- T_2

```
;$CLASS=HighRes
;$DIM=2D
```

```
;$TYPE=
;$SUBTYPE=
;$COMMENT=
prosol relations=<triple_na>
#include <Avance.incl>
#include <Grad.incl>
#include <Delay.incl>
"p2=p1*2"
"p22=p21*2"
"d11=30m"
"d26=1s/(cnst4*4)"
"d0=3u"
"in0=inf1/2"
"DELTA1=p16+d16+4u"
"DELTA3=d27-p2/2"
"DELTA=p16+d16+p2+d0*2"


"COUNTER=d26*2/((d21*2+larger(p2,p22))*16)"
"l4=COUNTER*16"
"d31=(d21*2+larger(p2,p22))*l4"

1 ze
;  d11 pl16:f3
;2 d1 do:f3
;3 50u pl3:f3 UNBLKGRAD
  d11 pl16:f2
2 d1 do:f2
3 50u pl3:f2 UNBLKGRAD
  20u rpp6
  20u rpp16
  (p1 ph1)
4 d21
  (center (p2 ph6) (p22 ph16):f2 )
  d21 ipp6 ipp16
```

```
  lo to 4 times l4
  (p1 ph2)
  4u
  p16:gp1
  d16
  (p21 ph3):f2
;;;;;;;;;;;; start T2
5          d27*2
  (p21 ph10 d27*2 p21 ph10 d27*2 p21 ph10 d27*2 p21 ph10):f2
  DELTA3
  (p2 ph1)
  DELTA3
  (p21 ph10 d27*2 p21 ph10 d27*2 p21 ph10 d27*2 p21 ph10):f2
  d27*2
  (p21 ph10 d27*2 p21 ph10 d27*2 p21 ph10 d27*2 p21 ph10):f2
  DELTA3
  (p2 ph1)
 DELTA3
  (p21 ph10 d27*2 p21 ph10 d27*2 p21 ph10 d27*2 p21 ph10):f2
   lo to 5 times l27

;;;;;;;;;;;; end T2



  d0
  (p2 ph5)
;  d0
  p16:gp2*EA
  d16
  (p22 ph4):f2
  DELTA
  (ralign (p1 ph1) (p21 ph4):f2 )
6 d21
  (center (p2 ph6) (p22 ph16):f2 )
  d21 ipp6 ipp16
```

```
  lo to 6 times l4

  DELTA1
  (p2 ph1)
  p16:gp3
  d16 pl16:f2
  4u BLKGRAD
  go=2 ph31 cpd3:f2
  d1 do:f2 mc #0 to 2
    F1EA(calgrad(EA), caldel(d0, +in0) & calph(ph3, +180) & calph(ph31, +180))
  d31
exit

ph1=0
ph2=1
ph3=0 2
ph4=0 0 0 0 2 2 2 2
ph5=0 0 2 2
ph6=0 1 0 1 1 0 1 0 2 3 2 3 3 2 3 2
ph10=0 0 0 0 2 2 2 2
ph16=0 1 0 1 1 0 1 0 2 3 2 3 3 2 3 2
ph31=0 2 0 2 2 0 2 0
;pl1 : f1 channel - power level for pulse (default)
;pl2 : f2 channel - power level for pulse (default)
;pl3 : f3 channel - power level for pulse (default)
;pl12: f2 channel - power level for CPD/BB decoupling
;pl16: f3 channel - power level for CPD/BB decoupling
;sp13: f2 channel - shaped pulse 180 degree (adiabatic)
;spnam13: Crp60,0.5,20.1
;l27: CPMG loop counter
;p1 : f1 channel -  90 degree high power pulse
;p2 : f1 channel - 180 degree high power pulse
;p8 : f2 channel - 180 degree shaped pulse for inversion (adiabatic)
;     = 500usec for Crp60,0.5,20.1
;p16: homospoil/gradient pulse                    [1 msec]
```

```
;p21: f3 channel -  90 degree high power pulse
;p22: f3 channel - 180 degree high power pulse
;d0 : incremented delay (2D)                    [3 usec]
;d1 : relaxation delay; 1-5 * T1
;d11: delay for disk I/O                        [30 msec]
;d16: delay for homospoil/gradient recovery
;d21: delay in XY16-CPMG                        [100 usec]
;d26: 1/(4J(PH))
;d31: 1/(2J(PH)) as executed
;cnst4: = J(PH)                                 [20 Hz]
;o2p: 77 ppm
;o3p: 0 ppm
;l4: loop for XY16-CPMG
;inf1: 1/SW(P) = 2 * DW(P)
;in0: 1/(2 * SW(P)) = DW(P)
;nd0: 2
;ns: 4 * n
;ds: >= 32
;td1: number of experiments
;FnMODE: echo-antiecho
;cpd2: decoupling according to sequence defined by cpdprg2
;cpd3: decoupling according to sequence defined by cpdprg3
;pcpd2: f2 channel - 90 degree pulse for decoupling sequence
;pcpd3: f3 channel - 90 degree pulse for decoupling sequence
;use gradient ratio:      gp 1 : gp 2 : gp 3
;                           50 :   80 : 32.4     for P-31
;for z-only gradients:
;gpz1: 10%
;gpz2: 80%
;gpz3: 32.4% for P-31
;use gradient files:
;gpnam1: SMSQ10.100
;gpnam2: SMSQ10.100
;gpnam3: SMSQ10.100
```

;$Id: na_hsqcetf3gpxy,v 1.6 2015/07/20 11:20:57 ber Exp $

References

1 Felli, I. C. & Pierattelli, R. Novel methods based on 13C detection to study intrinsically disordered proteins. *Journal of Magnetic Resonance* **241**, 115-125, doi:10.1016/j.jmr.2013.10.020 (2014).

2 Gil, S. *et al.* NMR spectroscopic studies of intrinsically disordered proteins at near-physiological conditions. *Angew Chem Int Ed Engl* **52**, 11808-11812, doi:10.1002/anie.201304272 (2013).

3 Rule, G. & Hitchens, K. *Fundamentals of Protein NMR Spectroscopy*. 1st edn, (Springer, Dordrecht, 2006).

4 Gascoigne, L. *et al.* Fractal-like R5 assembly promote the condensation of silicic acid into silica particles. *J Colloid Interface Sci* **598**, 206-212, doi:10.1016/j.jcis.2021.04.030 (2021).

5 Rubinstein, M. & Colby, R. H. *Polymer physics*. Vol. 23 (Oxford university press New York, 2003).

6 Daus, F., Xie, X. & Geyer, A. The silica mineralisation properties of synthetic Silaffin-1A(1) (synSil-1A(1)). *Org Biomol Chem* **20**, 3387-3396, doi:10.1039/d2ob00390b (2022).

7 Polyansky, A. A., Gallego, L. D., Efremov, R. G., Kohler, A. & Zagrovic, B. Protein compactness and interaction valency define the architecture of a biomolecular condensate across scales. *Elife* **12**, doi:10.7554/eLife.80038 (2023).

8 Polyansky, A. A., Gallego, L. D., Efremov, R. G., Köhler, A. & Zagrovic, B. in *bioRxiv* (bioRxiv, 2023).

9 Strobl, J. *et al.* Understanding Self-Assembly of Silica-Precipitating Peptides to Control Silica Particle Morphology. *Adv Mater*, e2207586, doi:10.1002/adma.202207586 (2022).
